# Steric Control of Molecular Packing Governs Excimer Emission in Fluorene-Based Molecular Solids

**DOI:** 10.1021/acs.chemmater.6c01251

**Published:** 2026-08-10

**Authors:** Carla Cunha, José A. Paixão, J. Sérgio Seixas de Melo

**Affiliations:** † CQC, Department of Chemistry, 37829 University of Coimbra, P3004-535 Coimbra, Portugal; ‡ CFisUC, Department of Physics, University of Coimbra, Rua Larga, Coimbra 3004-516, Portugal

## Abstract

For the rational design of solid-state organic luminophores, achieving predictable control over excimer emission remains a central challenge, as it is governed by subtle variations in molecular packing. Here, we demonstrate that excimer emission in fluorene-based molecular solids is a sterically programmable excited-state pathway that can be selectively enabled or suppressed through molecular design. A systematic series of fluorene derivatives bearing symmetric (2,7-dmF, 2,7-*dtb*F, and 3,6-*dtb*F) and asymmetric (2-*tb*F) alkyl substituents was investigated to elucidate how steric effects control supramolecular organization and excited-state behavior. Combined photophysical characterization, single-crystal X-ray diffraction, and DFT/TDDFT calculations establish a direct structure-packing-property relationship governing emission pathway. Planar fluorene and 2,7-dimethyl derivatives adopt antiparallel π–π stacked arrangements that stabilize excimer states, resulting in intense solid-state emission with high photoluminescence quantum yields (up to ∼60%) and nanosecond-scale lifetimes (up to ∼12 ns), accompanied by delayed components indicative of packing-controlled excimer formation. In contrast, *tert*-butyl substitution disrupts cofacial π–π interactions, suppressing excimer formation and yielding predominantly monomer-like emission. This steric control is further validated in fluorene: diamantane mixtures, where diamantane acts as a rigid three-dimensional spacer that increases intermolecular separation and inhibits excimer formation. Complementary dimer-based electronic structure calculations and charge-density analyses provide molecular-level insight into how steric constraints modulate intermolecular electronic coupling and excited-state character. These findings establish excimer emission as a packing-controlled and designable excited-state phenomenon and provide general design principles for tuning solid-state luminescence through steric engineering of molecular assemblies.

## Graphical abstract

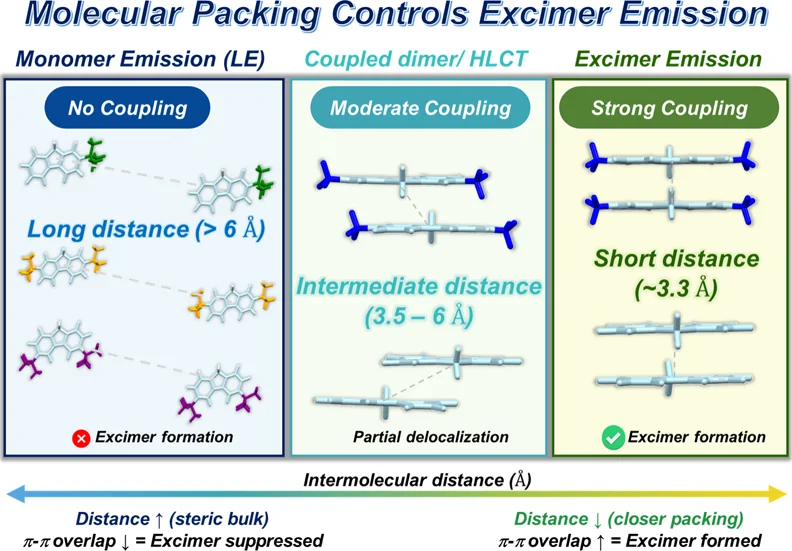

## Introduction

Fluorene and its derivatives are widely employed in optoelectronic applications owing to their rigid, planar structure, which supports efficient blue emission and favorable charge-transport properties.^1–12^ Functionalization of the fluorene core provides a versatile platform for modulating intermolecular interactions and solid-state organization, making these systems ideal model compounds for studying structure–property relationships in organic molecular solids. Beyond covalent modification, noncovalent interactions such as π–π stacking, C–H···π contacts, and weak hydrogen bonding can be exploited to control emission through supramolecular design.^13^

A key challenge in designing solid-state luminophores is achieving predictable control over excimer emission. This involves close π–π interactions in planar aromatic systems, where a single electronically excited molecule and one in the ground state give rise to red-shifted, broad emission bands distinct from monomer fluorescence, reflecting the formation of an excited-state dimer. Excimers are thus emissive excited-state dimers formed through short-range intermolecular electronic coupling. Excimers arise from intermolecular interactions involving electronically excited chromophores and are highly sensitive to molecular packing.^14–18^ While excimer emission is well documented in molecules such as pyrene, anthracene, and carbazole, its occurrence in the solid state remains difficult to control due to the subtle and often unpredictable nature of molecular packing.^19–22^ In addition, both the size and nature of substituent groups strongly influence excimer formation and stability.^23^

In crystalline solids, excimer formation is governed by the spatial arrangement and electronic coupling of neighboring chromophores, which depend on intermolecular interactions such as π–π stacking and van der Waals contacts.^24–31^ Empirically, efficient excimer formation typically requires interplanar separations of ∼3.3–3.8 Å together with favorable molecular orientation to maximize orbital overlap.^32–36^ However, the degree of slip and relative orientation can strongly modulate the intermolecular electronic coupling and the resulting excited-state character.

Despite extensive studies, excimer formation in organic solids is still frequently treated either as an intrinsic property of specific chromophores or as an undesirable consequence of aggregation. In many systems, strong intermolecular interactions lead to aggregation-caused quenching (ACQ),^35,37–40^ where excimers act as low-energy trap states with inefficient radiative decay.^38,41^ As a result, strategies in organic photophysics have traditionally focused on suppressing intermolecular interactions rather than controlling them. However, emerging evidence suggests that excimer emission can be exploited as a useful emissive pathway when intermolecular coupling is precisely controlled. This raises a central question: can excimer formation be rationally designed through control of molecular packing, rather than being treated as an unavoidable or detrimental phenomenon?

Here, we demonstrate that excimer emission in fluorene-based molecular solids is a sterically programmable excited-state pathway governed by molecular packing. Using a systematic series of fluorene derivatives with symmetric and asymmetric alkyl substitution patterns (2,7-dmF, 2,7-*dtb*F, 3,6-*dtb*F, and 2-*tb*F), we show that alkyl substitution (via steric effects) directly controls supramolecular organization and intermolecular electronic coupling, as shown in Scheme 1. By combining photophysical characterization, single-crystal X-ray diffraction, and DFT/TDDFT calculations, we establish a clear structure-packing-excited-state relationship that determines whether excimer emission is enabled or suppressed. We demonstrate that excimer formation is not an intrinsic molecular property, but rather a packing-dependent phenomenon that can be selectively controlled through steric modulation of intermolecular contacts, while the monomer absorption/emission signatures remain essentially unchanged in dilute solution. These findings establish excimers as designable excited states in molecular solids and provide general design principles for tuning solid-state luminescence through steric modulation of intermolecular interactions.

**Scheme 1:**
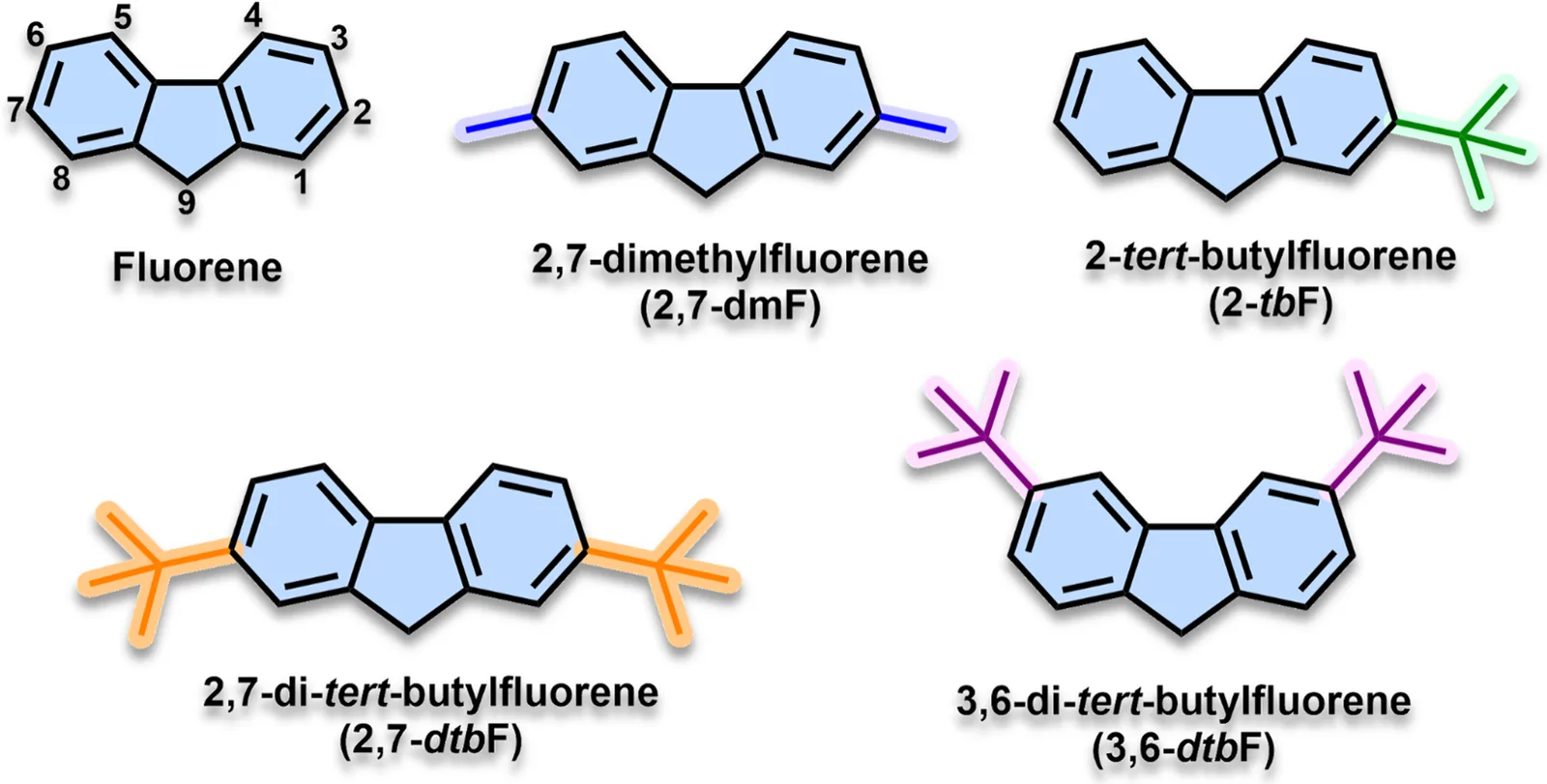
Chemical Structures (With Numbering in Fluorene) and Acronyms of the Fluorene Derivatives

## Experimental Section

### Materials and Instrumentation

Chemicals were purchased from Sigma-Aldrich, ABCR GmbH or TCI chemicals and used without further purification, unless described otherwise. For photophysical experiments, solutions were prepared using spectroscopic-grade solvents or equivalent. Deoxygenation was done by bubbling the solutions with a stream of argon or nitrogen for approximately 20 min in a device elsewhere described.^42^ All measured solutions were freshly prepared (within the day) unless noted otherwise.

### Photophysical Measurements

Steady state and time-resolved photoluminescence. For the photophysical experiments, all solvents used were of spectroscopic or equivalent grade. Absorption spectra were obtained in a 10 mm quartz cuvette in different organic solvents on a Shimadzu UV-2600 spectrophotometer or Agilent Cary 5000 UV–vis–NIR spectrometers (thin films and powders). Fluorescence spectroscopic studies were performed using a Horiba–Jobin–Yvon Fluorolog 3.22. Fluorescence spectra were corrected for the wavelength response of the system. Fluorescence quantum yields in solution were measured at room temperature using naphthalene (in cyclohexane, ϕ_F_ = 0.23)^43^ as standard. Solid-state fluorescence quantum yields (thin films, powders, and crystals) and fluorescence and phosphorescence quantum yields at 77 K were measured using an absolute method with a Hamamatsu Quantaurus QY absolute photoluminescence quantum yield spectrometer, model C11347 (integration sphere). A clean sapphire substrate was used as reference for the ϕ_F_ measurements of solid-state. Fluorescence decays were obtained either in aerated or degassed solutions and were measured with two different custom-built time-correlated single-photon counting equipment (TCSPC).^44,45^ Fluorescence decay measurements of the fluorene derivatives, both in solution and in thin films, were carried out using a home-built picosecond time-correlated single photon counting (ps-TCSPC) apparatus, as described elsewhere.^45^ The excitation source consists of a tunable picosecond Spectra-Physics mode-lock Tsunami laser (Ti: Sapphire) model 3950 (80 MHz repetition rate, tuning range 700–1000 nm), pumped by a 532 nm continuous wave Spectra-Physics Millennia Pro-10s laser. The excitation wavelength (λ_exc_ = 268 nm) was obtained with a Spectra-Physics harmonic generator, model GWU-23PS. To eliminate the low quantity of dispersed light, an RG530 filter was used after the sample holder and before the emission monochromator. Temperature control was achieved using a home-built system based on cooled nitrogen and electric heating. For fluorene-based derivatives in the powder form, ns-TCSPC measurements were performed following previously procedures, with the modification that an IBH nanoLED (excitation at 282 nm) was employed as the excitation source.^44^ The fluorescence decay curves were deconvoluted using the experimental instrument response function signal collected with a scattering solution (aqueous Ludox solution). The deconvolution procedure was performed using the modulation function method, as implemented by G. Striker in the SAND program, and previously reported in the literature.^46^

### Film Preparation

Thin films from the compounds were obtained with a desktop precision spin-coating system, model P6700 series from Speedline Technologies as elsewhere described.^47^ Briefly, thin films from the samples were obtained by deposition of ca. 50 μL from a solution of the compounds into a circular sapphire substrate (12 mm diameter) followed by spin-coating (2500 rpm for 60 s) in a nitrogen-saturated atmosphere (2 psi). The solutions for spin-coating were prepared by adding 2 mg of the samples along with 15 mg of Zeonex to 200 μL of chloroform solution. Before the film deposition procedure was made, the solutions were stirred overnight at room temperature.

### X-ray Diffraction

Single crystals suitable for X-ray diffraction analysis were grown for 2,7-*dtb*F and 3,6-*dtb*F by slow diffusion in an ethanol solution of the compound. The crystal structure of unsubstituted fluorene discussed below was taken from the literature. However, suitable single crystals for structural analysis could not be obtained for 2,7-dimethylfluorene (2,7-dmF) and 2-*tert*-butylfluorene (2-*tb*F). In the case of unsubstituted fluorene, only thin needle-like crystals were obtained, which were not suitable for single-crystal X-ray diffraction due to insufficient diffraction quality. Consequently, the crystallographic analysis presented herein is limited to the representative derivatives 2,7-*dtb*F and 3,6-*dtb*F, for which high-quality single crystals were successfully obtained. The crystal data and experimental details for the data collections are given below. Single crystal X-ray data for 2,7-*dtb*F and 3,6-*dtb*F were collected at room temperature using graphite monochromated MoKα (λ = 0.71073 Å) radiation on a Bruker APEX II diffractometer equipped with a 4K CCD detector. Integration and correction for Lorentz and polarization factors were performed using SAINT V8.38A^48^ included in the APEXIII package.^49^ Absorption corrections, including odd and even spherical harmonics up to rank 3 and 6, respectively, were performed using SADABS-2016/2.^50^ The structure was solved by the dual-space algorithm implemented in SHELXT-2018/2,^51^ and the structural model was refined using the full-matrix least-squares method implemented in SHELXL-2019/3.^51^ All non-hydrogen atoms were refined anisotropically. Hydrogen atoms were placed at calculated idealized positions and refined as riding using SHELXL-2019/3 default values. Both compounds crystallize in the monoclinic system, space groups *I2/c* (#15, 2,7-*dtb*F) and *P2*_1_/*c* (#14, 3,6-*dtb*F).

The crystal 2,7-*dtb*F used for the X-ray data collection turned out to be a two-component twin (structure is monoclinic with cell metrics being close to orthorhombic, the twin law corresponding to a 2-fold rotation along the *c*-axis), and the structure was refined accordingly. Structure validation was performed with PLATON, ORTEP illustration and drawing of packing diagrams were performed with PLATON and Mercury. Crystallographic details are given in Tables S4 and S5.

In compound 3,6-*dtb*F, one of the *tert*-butyl groups is disordered over two staggered conformations, with 77%/23% occupancy probability in the crystal. Bond lengths and valency angles are unexceptional. In both 2,7-*dtb*F and 3,6-*dtb*F, the fluorene moiety is planar (maximum and rms deviation of the least-squares plane of the rings: 2,7-*dtb*F: 0.04 Å, 0.02 Å; 3,6-*dtb*F: 0.04 Å, 0.02 Å). The structures contain no solvent accessible voids. As the structures do not feature classical hydrogen bonds, cohesion of the crystals is determined by van der Waals interactions and Cg···Cg and C–H···Cg interactions involving the π electron clouds, described further below. CIF files containing the supplementary crystallographic data were deposited at the Cambridge Crystallographic Data Centre, with references CCDC 2454231 (2,7-*dtb*F) and CCDC 2454297 (3,6-*dtb*F).

### Quantum Electronic Calculations

All theoretical calculations were performed using density functional theory (DFT) and time-dependent DFT (TDDFT) as implemented in the GAMESS-US version R3.^52^ Ground- and excited-state calculations were carried out using the range corrected LC-BPBE (ω = 0.20 au^–1^), which includes long-range exchange correction and is suitable for describing charge-transfer and spatially extended excited states.

A 6-31G** basis set was employed for both DFT and TDDFT calculations as a balanced compromise between computational cost and accuracy for the relatively large π-stacked dimer systems considered in this work. Although diffuse functions are often recommended for highly delocalized or Rydberg-type excited states, test calculations on representative dimers showed no qualitative changes in the nature or ordering of the relevant low-lying excited states upon their inclusion, supporting the adequacy of the chosen basis set for the present systems.

Excited-state properties and potential energy surfaces (PES) were explored using TDDFT calculations with the same functional and basis set. Vertical electronic excitation energies were obtained for both monomeric and dimeric structures, and all reported values correspond to unscaled raw data. Natural transition orbitals (NTOs) and molecular orbital contours were used to analyze the character of the electronic excitations. Molecular orbital contours were plotted using the ChemCraft 1.7 program.

A solvent was included using the polarizable continuum model (PCM) combined with the solvation model density (SMD) to add corrections for cavitation, dispersion, and solvent structure. In TDDFT calculation of Franck–Condon excitations, the dielectric constant of the solvent was split into a “bulk” component and a fast component, which is essentially the square of the refractive index. Under adiabatic conditions, only the static dielectric constant was employed.

Geometry optimizations were performed at the same level of theory for both ground and excited states. Frequency analysis for each compound was also computed and did not yield any imaginary frequencies, indicating that the structure of each molecule corresponds to at least a local minimum on the potential energy surface.

## Results and Discussion

### Structure-Dependent Photophysical Behavior in Solution and the Solid State

The photophysical properties of fluorene-based molecular solids were investigated in solution and in the solid state (thin films, single crystals, and powders), with absorption and photoluminescence (PL) spectra shown in Figure 1 and key parameters summarized in Table 1. This comparative approach isolates intrinsic molecular properties from packing-induced effects, establishing a benchmark for understanding excimer formation in the solid state.

**Figure 1:**
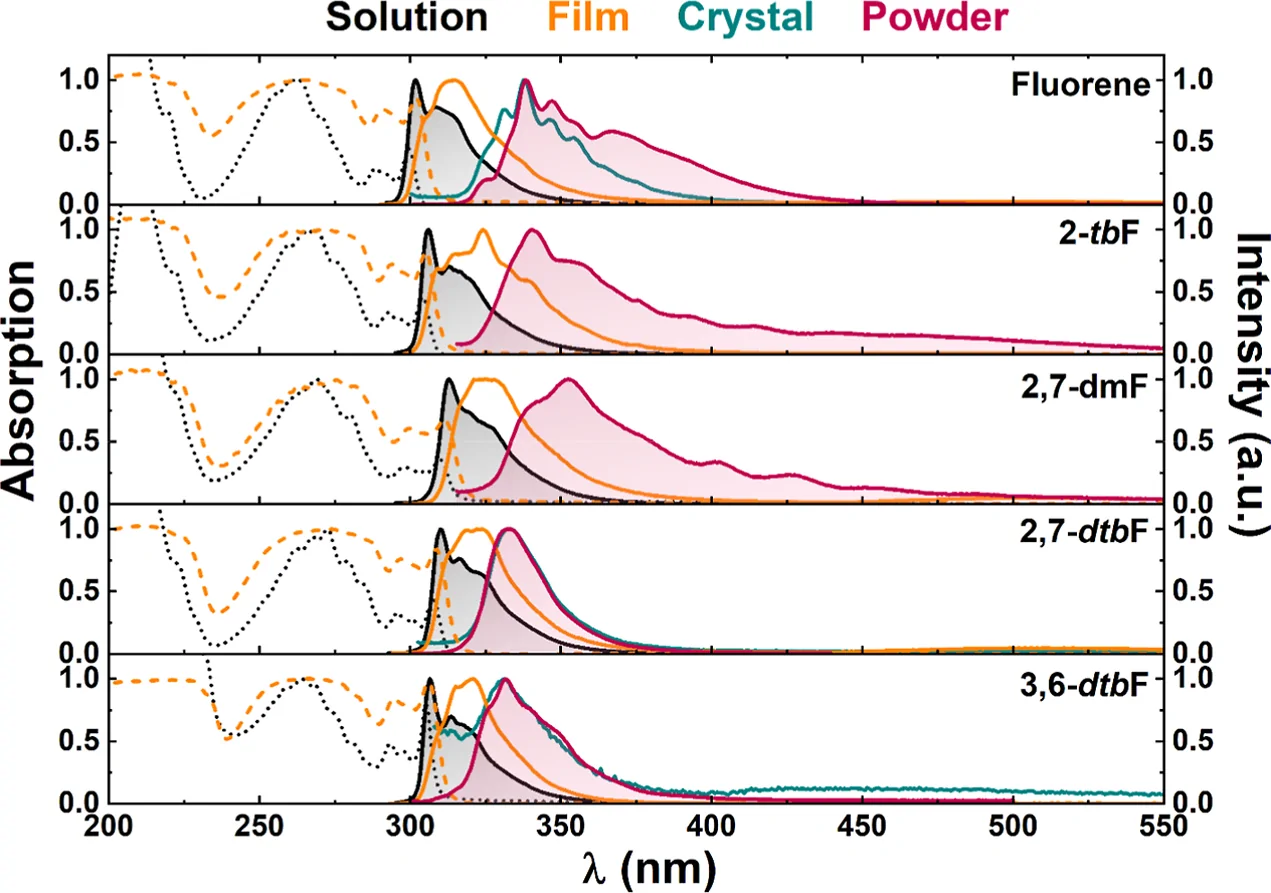
Normalized absorption and fluorescence emission spectra of fluorene derivatives recorded in cyclohexane (solution) and in the solid state (thin films, crystals, and powders) at *T* = 293 K. Fluorescence spectra were acquired with excitation at λ_exc_ = 290 nm.

**Table 1:** Photophysical Data of Fluorene Derivatives (including Wavelength Absorption, λ_abs_, Fluorescence Emission Maxima, λ_em_, and Stokes Shift, Δ_SS_ (cm^–1^)) in Solution (Cyclohexane) at 293 K and Phosphorescence Maximum (λ_Phosp_) in Methylcyclohexane at 77 K and Solid State (Thin Films, Crystals, and Powders)

compound	medium	λ_abs_ (nm)	λ_em_ (nm)	Δ_SS_ (cm^–1^)	λ_Phosp_ (nm)	ϕ_F_
fluorene	solution	261	302	5202	422	0.496
	film	267	315	5707	-	0.138
	crystal	-	338	-	-	0.413
	powder	330	338	717	-	0.655
2,7-dmF	solution	269	313	5226	463	0.512
	film	274	325	5727	-	0.177
	powder	311	353	3826	-	0.616
2-*tb*F	solution	269	306	4495	457	0.500
	film	271	324	6036	-	0.083
	powder	325	341	1444	-	0.467
2,7-*dtb*F	solution	273	310	4372	459	0.536
	film	274	324	5632	-	0.100
	crystal	-	332	-	-	0.402
	powder	309	332	2242	-	0.520
3,6-*dtb*F	solution	264	307	5305	456	0.540
	film	266	321	6441	-	0.091
	crystal	-	331	-	-	0.075
	powder	305	332	2666	-	0.070

In dilute solution, all compounds exhibit nearly identical optical behavior, characterized by structured absorption (261–273 nm) and emission (302–313 nm) bands assigned to localized π–π* transitions on the fluorene core. TDDFT calculations confirm that the lowest excited states are fluorene-centered, with negligible orbital contribution from the substituents. Consistent with this assignment, minimal solvatochromism is observed across solvents ranging from cyclohexane (ε′ = 2.023) to acetonitrile (ε′ = 35.94), as shown in Table S1, indicating minimal charge-transfer character.

Alkyl substitution induces systematic bathochromic shifts in both absorption and emission maxima relative to unsubstituted fluorene, following the trend fluorene <2-*tb*F < 3,6-*dtb*F < 2,7-*dtb*F < 2,7-dmF. These shifts arise from inductive effects that modulate the local electronic environment of the fluorene core and stabilize the excited states without altering their fundamental character. Accordingly, all derivatives display characteristic polycyclic aromatic hydrocarbon behavior in solution, with well-resolved vibronic structure, high fluorescence quantum yields, and minimal nonradiative decay (Tables 1 and S2).

In contrast, pronounced differences emerge in the solid state, highlighting the dominant role of intermolecular interactions. Thin films prepared by dispersing the fluorene derivatives in an optically inert Zeonex matrix provide a reference system in which intermolecular coupling is effectively suppressed. Under these conditions, absorption spectra closely resemble those in solution, exhibiting only minor shifts (Δλ = 1–6 nm), whereas emission spectra show moderate bathochromic shifts (12–18 nm) and spectral broadening, accompanied by slight increases in Stokes shifts (by a factor of 1.1–1.3). These features are consistent with weak, nonspecific intermolecular interactions in the absence of well-defined packing motifs.

Crucially, the absence of additional low-energy emission bands in Zeonex films demonstrates that excimer formation is effectively suppressed when chromophores are spatially isolated. Emission therefore remains predominantly monomeric, defining a well-characterized reference state that reflects the intrinsic photophysics of the fluorene core in the absence of strong π–π interactions. This provides an essential baseline for evaluating how controlled molecular packing in crystalline phases promotes excimer formation. Together, the solution and Zeonex data establish that the substituents have minimal effect on the monomer electronic structure, enabling solid-state spectral changes to be attributed primarily to packing-controlled intermolecular coupling.

### Solid-State Photophysics and Excimer Formation: Correlation between Emission and Crystal Packing

To establish a direct link between molecular packing and excited-state behavior, crystalline samples of fluorene, 2,7-*dtb*F, and 3,6-*dtb*F were investigated. In contrast to solution and polymer-diluted thin films, these compounds exhibit pronounced changes in their emission profiles, reflecting the emergence of strong intermolecular electronic coupling in the solid state.

Crystalline samples are found to display significantly broadened and red-shifted emission bands, consistent with excimer formation driven by intermolecular π–π interactions. The extent of this spectral transformation varies systematically with substitution pattern, indicating that steric effects modulate intermolecular overlap and excimer stabilization efficiency. In particular, differences between 2,7- (2,7-*dtb*F) and 3,6-substitution (3,6-*dtb*F) highlight how subtle variations in steric demand influence packing geometry and control access to excimer-forming conformations.

Although single-crystal structures were not obtained for all derivatives, the available crystallographic data provide critical insight into the relationship between steric substitution and molecular organization. Variations in emission profiles and excited-state decay dynamics correlate directly with differences in packing efficiency and intermolecular contact, supporting a model in which excimer formation is governed by the extent of π–π overlap imposed by crystal packing. These results demonstrate that excimer emission in fluorene-based solids is not dictated by molecular identity alone, but arises from sterically controlled packing motifs that regulate intermolecular electronic coupling.

### Solid-State Emission in Powder Samples

In powder form, the photoluminescence behavior of the fluorene derivatives deviates markedly from that observed in solution, reflecting the increasing influence of intermolecular interactions in the condensed phase. All compounds exhibit solid-state fluorescence with emission maxima red-shifted by 22–40 nm relative to solution.

For fluorene and 2,7-dmF, the powders display pronounced bathochromic shifts accompanied by broad, weakly structured emission bands and loss of vibronic resolution, consistent with the formation of intermolecularly coupled excited states. These spectral characteristics are indicative of excimer-like emission arising from efficient π–π interactions between closely packed fluorene units. In contrast, the *tert*-butyl substituted derivatives (2-*tb*F, 2,7-*dtb*F and 3,6-*dtb*F) exhibit broader but less red-shifted emission that remains clearly distinct from the excimer-like spectra of fluorene and 2,7-dmF, reflecting weaker intermolecular electronic coupling imposed by steric hindrance.

To further distinguish excimer emission from alternative aggregate-related emissive states, excitation-wavelength-dependent emission spectra were recorded for all powder samples (Figure S1). Over the investigated excitation range (290–309 nm), all derivatives exhibit essentially identical emission profiles, with only variations in emission intensity. The absence of excitation-wavelength-dependent spectral changes demonstrates that the broad solid-state emission originates from a common relaxed emissive state rather than from multiple selectively excited aggregate populations.

### Photoluminescence Properties and Excited-State Dynamics of Fluorene Derivatives

Quantitative analysis of the photophysical parameters (Table 1) supports the existence of distinct regimes of intermolecular electronic coupling in the fluorene derivatives. In particular, the Stokes shift (Δ_SS_), fluorescence quantum yields (ϕ_F_), and time-resolved fluorescence measurements demonstrate that excited state relaxation evolves from localized monomer emission in solution to packing-dependent excited-state processes in the solid-state.

Time-resolved fluorescence measurements with nanosecond resolution were performed in dilute solution, thin films, and powder samples to elucidate how substitution pattern and solid-state organization govern the excited-state dynamics of the fluorene derivatives (see Supporting Information for further details). Representative decay profiles are shown in Figure 2, while the radiative (*k*_R_) and nonradiative (*k*_NR_) rate constants are summarized in Table S2. The decay traces recorded at multiple emission wavelengths were analyzed using a global fitting procedure, enabling the extraction of common kinetic components associated with the distinct emissive populations. Consequently, the lifetime components are interpreted as reflecting excited-state processes that govern the emissive behavior throughout the different wavelengths.

**Figure 2:**
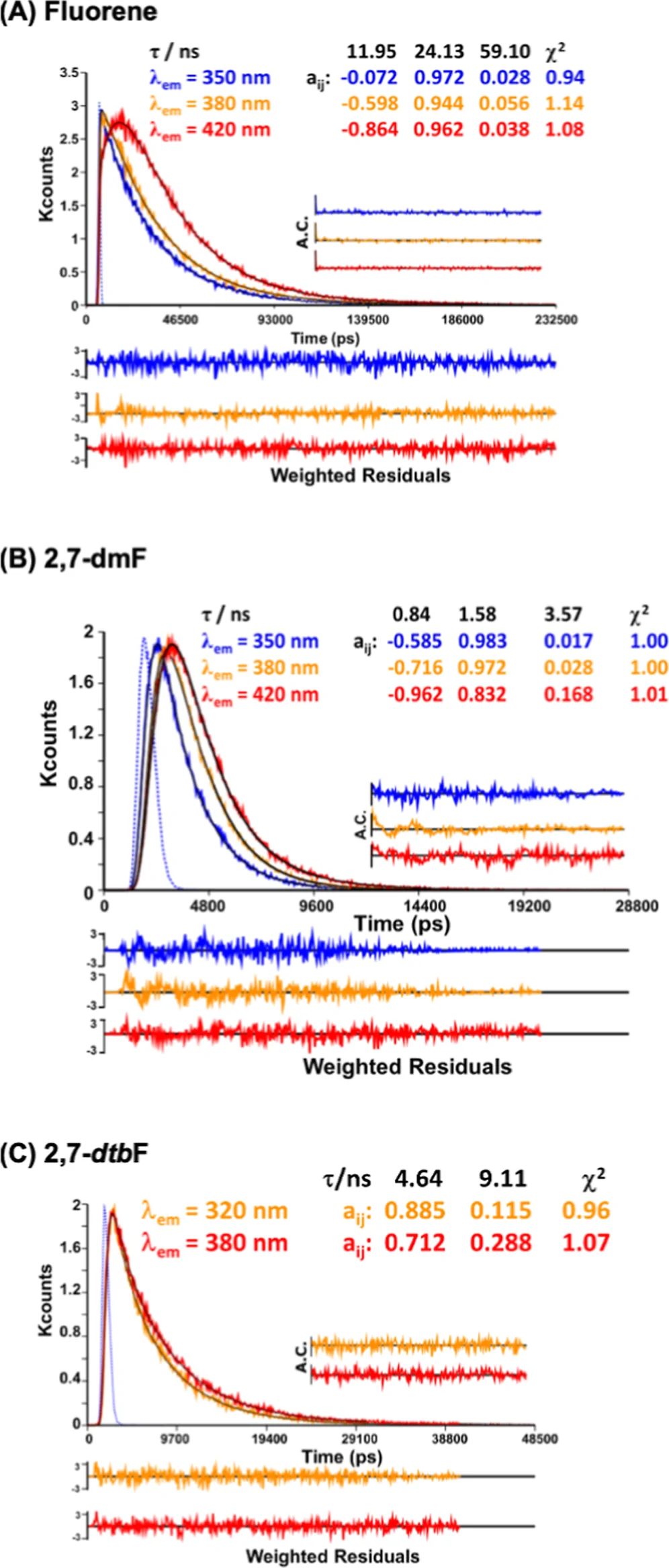
Room temperature fluorescence decays for fluorene derivatives in powder. For a better judgment of the quality of the fit, weighted residuals (W.R.), autocorrelation function (A.C.) and χ^2^ values are also presented. The dashed line in the decay corresponds to the instrumental response function.

In dilute solution, all compounds exhibit single-exponential fluorescence decays with lifetimes between 3.75 and 4.97 ns, Table S2 and Figure S2, showing only a weak dependence on solvent polarity. The fluorescence quantum yields remain uniformly high (ϕ_F_ = 0.47–0.54), indicating that emission originates from localized fluorene-centered S_1_ states with negligible intermolecular interactions. Derivatives bearing *tert*-butyl substituents (2-*tb*F, 3,6-*dtb*F, and 2,7-*dtb*F) display slightly shorter lifetimes than unsubstituted fluorene, while other substitution patterns have minimal influence.

In Zeonex films, the fluorescence decays are best described by a biexponential model consisting of a short-lived component (τ_1_ = 1.31–1.92 ns) and a longer-lived component (τ_2_ = 3.15–4.89 ns), see Table S3 and Figure S3. The appearance of two kinetic components reflects the presence of different local molecular environments within the polymer matrix rather than distinct emissive species. The dominant longer-lived component is attributed to isolated monomer-like emitters, whereas the shorter component is assigned to fluorene units involved in weak intermolecular interactions or local packing heterogeneity. Compared with solution, the fluorescence quantum yields decrease substantially (ϕ_F_ = 8–18%), indicating that matrix-induced nonradiative decay pathways become more competitive despite the suppression of strong chromophore–chromophore interactions.

A markedly different behavior is observed in crystalline powders, where fluorescence decays becomes more complex due to the coexistence of emissive sites exhibiting varying degrees of intermolecular electronic coupling. Consequently, the multiexponential decay components should be regarded as kinetic descriptors of distinct local packing environments rather than discrete emissive species. The relative amplitudes of the individual components further reflect the fraction of excited-state populations associated with each emissive environment.

For fluorene and 2,7-dmF, the decay profiles display negative pre-exponential amplitudes associated with the shortest lifetime component, corresponding to rise times of approximately 12 and 0.84 ns, respectively. Importantly, the magnitude of the negative amplitude increases systematically with increasing emission wavelengths, where the excimer emission band becomes dominant. This wavelength dependence provides direct kinetic evidence for delayed excimer formation following photoexcitation. The dominant intermediate lifetime, accounting for more than 70% of the total emissive contribution, is assigned to the principal emissive excimer population, whereas the minor longest-lived component most likely reflects a small fraction of excimer sites located in slightly more stabilized local packing environments. The excellent quality of the global fits (χ^2^ ≈ 1) further supports the robustness of this kinetic model.

Although both fluorene and 2,7-dmF form emissive excimers, their excited-state relaxation differs significantly. Fluorene displays a small Stokes shift (717 cm^–1^), a long excimer lifetime (∼12 ns), and a high ϕ_F_ (0.655), consistent with a relatively rigid intermolecularly excited state undergoing limited structural relaxation. In contrast, 2,7-dmF exhibits a significantly larger Stokes shift (3826 cm^–1^) together with a considerably shorter excimer lifetime (0.84 ns), indicating increased excited-state structural relaxation while maintaining a similarly high fluorescence efficiency. These observations suggest that efficient excimer emission can arise from different relaxation regimes depending on the molecular packing geometry imposed by substitution. Notably, the high solid-state ϕ_F_ values observed for fluorene and 2,7-dmF demonstrate that excimer formation does not inherently lead to aggregation-caused quenching (ACQ), but can instead result in efficient radiative decay when molecular packing promotes an emissive excimer geometry.

In contrast, none of the *tert*-butyl-substituted derivatives exhibit rise-time components, indicating that excimer formation is not established under these packing conditions. Their multiexponential decays instead attributed to heterogeneous monomer-like excited states associated with different local intermolecular interactions. For 2-*tb*F and 2,7-*dtb*F, the fluorescence lifetimes remain comparable to those measured in solution, suggesting that intermolecular interactions perturb the excited-state energetics without stabilizing fully developed excimer states. Conversely, 3,6-*dtb*F exhibits consistently low ϕ_F_ values (∼0.07) despite similar lifetimes, indicating that steric congestion suppresses both efficient excimer formation and radiative recombination by preventing favorable π–π overlap and promoting nonradiative decay (Figure S4).

Further insight into the excited-state dynamics was obtained from wavelength-resolved TCSPC measurements. The observed fluorescence decay times, on the order of a few nanoseconds, are characteristic of prompt fluorescence from singlet excited states. Variations in both the spectral profiles and decay kinetics reflect different degrees of intermolecular electronic coupling, forming a continuum ranging from weakly coupled monomer-like emission to stabilized excimer states, depending on the extent of π–π interactions.

Global analysis of the wavelength-resolved TCSPC decays enabled reconstruction of the corresponding time-resolved emission spectra (TRES) for all powder samples. Representative examples for fluorene and 3,6-*dtb*F, which exhibit markedly different excited-state behavior, are presented in Figure S5. For fluorene, the normalized TRES reveal a subtle temporal redistribution of emission intensity during the initial delay times, characterized by a gradual increase in the long-wavelength contribution before the spectral profile becomes essentially constant. This behavior is fully consistent with the wavelength-dependent rise-time components identified by the global lifetime analysis and reflects excited-state relaxation toward a stabilized excimer within the preorganized molecular packing. In contrast, 3,6-*dtb*F exhibits nearly overlapping normalized TRES throughout the entire decay, with no measurable spectral evolution. Together with the absence of wavelength-dependent rise-time components, this behavior indicates that emission originates predominantly from weakly coupled monomer-like excited states rather than from emissive excimers.

These complementary steady-state and time-resolved measurements demonstrate that efficient excimer emission in fluorene-based solids is achieved only when the crystal packing enables strong intermolecular electronic coupling. When steric substitution disrupts this packing motif, excited-state relaxation proceeds without excimer stabilization, leading instead to localized monomer-like emission despite the presence of intermolecular contacts. Overall, the excited-state dynamics of these systems are governed by a continuum of intermolecular coupling regimes ranging from localized monomer emission to stabilized excimer states, where emission is determined not simply by the presence of intermolecular interactions but by the specific packing geometry that controls the formation of a kinetically well-defined emissive excimer population. This interpretation is further corroborated by structural analysis and theoretical calculations, which reveal mixed local-excitation and charge-transfer character in the S_1_ state arising from π-stacked fluorene units. Together, the experimental and computational results establish excimer formation as a packing-dependent excited-state pathway that can be systematically tuned through steric molecular design.

### Triplet-State Characterization

To unambiguously determine the origin of the long-wavelength emission observed in the solid state, the triplet excited states of the fluorene derivatives were investigated. Phosphorescence (T_1_ → S_0_) spectra were recorded in methylcyclohexane at low temperature (77 K) using delayed acquisition (delay after flash, DAF = 0.5 ms). Under these conditions, all derivatives exhibit nearly identical phosphorescence spectra, characterized by well-resolved vibronic structure in the 420–500 nm region and complete independence from excitation wavelength, Figure S6. These emissions are assigned to the lowest triplet excited state of predominant ^3^π,π* character localized on the fluorene core. The observed long phosphorescence lifetimes are consistent with spin-forbidden emission from rigid aromatic systems.

Only minor substituent effects are observed: *tert*-butyl derivatives (2,7-*dtb*F and 3,6-*dtb*F) display slight bathochromic shifts and modest variations in phosphorescence lifetimes relative to unsubstituted fluorene, reflecting subtle stabilization of the triplet state. Importantly, both the spectral shape and energy remain essentially unchanged across the series, demonstrating that alkyl substitution does not significantly perturb the nature or localization of the triplet excited state.

A comprehensive summary of the photophysical parameters is provided in Table 2. All derivatives exhibit high fluorescence quantum yields at 77 K (ϕ_F_ = 0.69–0.78) together with significant phosphorescence contributions (ϕ_Phosp_ = 0.13–0.22), resulting in combined radiative yields (ϕ_F_ + ϕ_Phosp_) ranging from 0.84 to 0.97. These values indicate that nonradiative decay pathways are largely suppressed under rigid conditions, and that excited-state deactivation is dominated by radiative processes. Notably, both the magnitude and distribution of radiative decay between singlet and triplet channels remain broadly consistent for the series, confirming that alkyl substitution has minimal impact on the intrinsic excited-state energetics of the fluorene chromophore.

**Table 2:** Photophysical Parameters (including Quantum Yields and Lifetimes) for the Fluorene and its Derivatives in Methylcyclohexane at Low Temperature (77 K)

compound	ϕ_F_ (77 K)	ϕ_Phosp_ (77 K)	ϕ_F_ + ϕ_Phosp_ (77 K)	τ_F_ (293 K), ns	τ_Phosp_ (77 K), ms
fluorene	0.738	0.194	0.932	4.66	2201
2,7-dmF	0.775	0.185	0.960	4.48	1.138
2-*tb*F	0.685	0.218	0.903	4.23	0.635
2,7-*dtb*F	0.763	0.206	0.969	3.95	3519
3,6-*dtb*F	0.707	0.130	0.837	3.98	1951

Crucially, the phosphorescence spectra differ markedly in both energy and vibronic structure from the long-wavelength emission observed in solid-state fluorescence at room temperature. While the triplet manifold remains essentially unchanged, the solid-state emission behavior varies dramatically as a function of substitution pattern and molecular packing. This clear decoupling demonstrates that the red-shifted emission observed in the solid state does not originate from triplet-state phosphorescence. Instead, the distinct spectral position, vibronic structure, and excited-state dynamics of the long-wavelength emission strongly assign it to singlet-state excimer formation.

Taken all together, these results establish that the emissive regimes of fluorene derivatives are intrinsically conserved at the molecular level, and that the emergence of additional emissive states in the solid state arises exclusively from packing-dependent intermolecular electronic coupling. This provides clear evidence that excimer emission is a packing-dependent phenomenon, rather than a consequence of intrinsic molecular electronic structure.

### Correlation between Crystal Structure and Solid-State Emission Behavior

Single-crystal X-ray diffraction studies were performed to establish the relationship between molecular packing and the solid-state photophysical behavior of the fluorene derivatives. Suitable single crystals were obtained for 2,7-*dtb*F and 3,6-*dtb*F, while the crystal structure of unsubstituted fluorene was taken from the literature for comparison.^53–55^

According to the reported structure, unsubstituted fluorene crystallizes in the orthorhombic *Pnma* space group with four molecules per unit cell.^53–55^ The crystal packing is characterized by well-defined face-to-face π–π interactions, with an interplanar distances of 3.5 Å and a centroid-to-centroid separation of 4.7 Å (intermolecular distance), Table 3. This nearly cofacial antiparallel arrangement maximizes intermolecular π-orbital overlap, providing a packing geometry favorable for strong intermolecular electronic coupling and excimer stabilization. Consistent with this structural motif, fluorene exhibits intense red-shifted solid-state emission together with wavelength-dependent rise-time components in the TCSPC measurements, providing direct evidence that the crystal packing governs the excited-state relaxation pathway.

**Table 3:** Displacements of the Dimer in Crystal and Optimized Ground-State Dimer Geometry Along With a, b and c Direction (a: Perpendicular to Fluorene plane, b: Along With the Short Axis of Fluorene unit, *c*: Along With the Long Axis of Fluorene Unit) and the Tendency of Excimer Formation

compound	interplanar distance (Å)^a^	intermolecular distance (Å)^b^	intermolecular distance (Å)^c^	excimer formation
fluorene “A”	3.478; 2.531	4.691	4.487	yes
fluorene “B”	-	5.717	5.717	no
fluorene “C”	-	9.905	9.758	no
2,7-*dtb*F	5.356; 4.437	9.175; 10.159	9.121	no
3,6-*dtb*F	3.627; 3.473	4.231; 6.924	4.018; 6.604	no

a Distances between the average planes of the molecules, see Figure 3, left panel.

b Intermolecular distances between the centroid of mass of the molecules, see Figure 3, right panel.

c Intermolecular distances between the centroid of mass of the molecules predicted by DFT theoretical calculations in the gas phase (ground state).

Single crystals suitable for structural analysis were obtained for 2,7-*dtb*F and 3,6-*dtb*F by slow evaporation from ethanol, Figure S7, Tables S4 and S5. Comparison of these structures reveals the pronounced influence of steric substitution on molecular packing. In both derivatives, the bulky *tert*-butyl groups disrupt the cofacial packing observed for fluorene, producing slipped or offset molecular arrangements with larger intermolecular separations and reduced π-orbital overlap (Table 3 and Figure 3). Although intermolecular contacts are preserved, the unfavorable relative orientation of neighboring fluorene units substantially weakens intermolecular electronic coupling. Consequently, these packing motifs do not efficiently stabilize excimer states, and the emission remains dominated by weakly coupled dimeric or monomer-like excited states, in agreement with the steady-state and time-resolved spectroscopic results.

**Figure 3:**
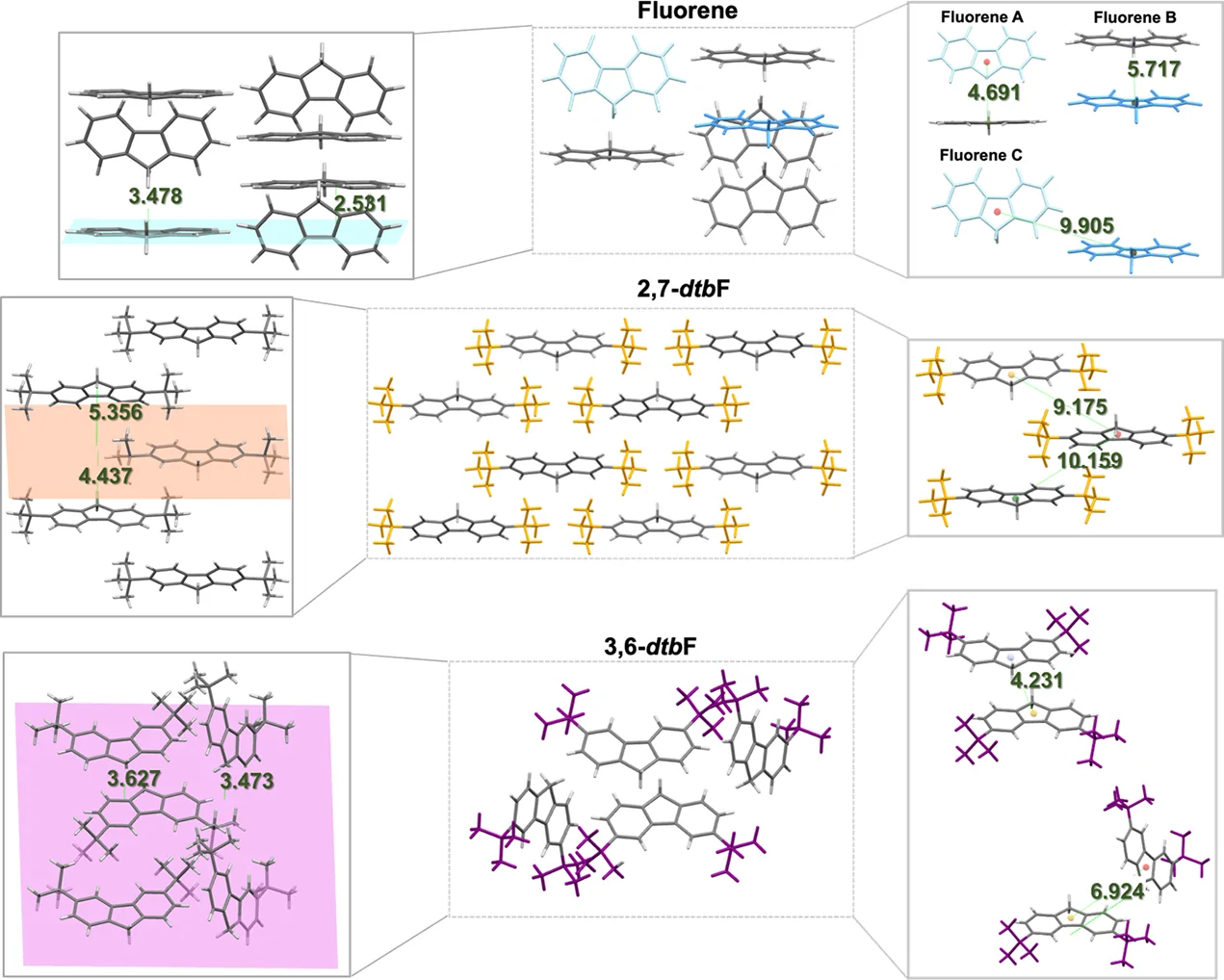
Crystal packing arrangements and representative intermolecular contacts of fluorene (data from crystal from ref 54), 2,7-*dtb*F and 3,6-*dtb*F. For fluorene, the asymmetric unit contains three crystallographically independent molecules, denoted Fluorene A, Fluorene B, and Fluorene C. The left-hand panels show the local packing environment of each compound, whereas the right-hand panels highlight representative intermolecular contacts relevant to the discussion. Green dashed lines indicate the shortest intermolecular contacts, with the corresponding distances given in Å. Carbon atoms are shown in gray, while the *tert*-butyl substituents are highlighted using different colors for clarity.

Importantly, the present results demonstrate that intermolecular distance alone cannot account for the observed emission behavior. Instead, excimer stabilization depends on the combined effects of intermolecular separation, relative molecular orientation, and the extent of cofacial π-orbital overlap, which together determine the magnitude of intermolecular electronic coupling. Small steric modifications therefore alter not only the distance between neighboring fluorene units but also their mutual orientation, shifting the excited-state relaxation pathway from efficient excimer formation toward weakly coupled monomer-like emission.

Although single-crystal structures were not obtained for all derivatives, the representative crystal structures, together with the systematic photophysical trends observed across the molecular series, establish a consistent structure–property relationship. The excimer emission observed for fluorene and 2,7-dmF is associated with packing arrangements that maximize intermolecular electronic coupling, whereas the *tert*-butyl-substituted derivatives illustrate how steric congestion progressively suppresses excimer stabilization by disrupting the packing geometry while preserving only weak intermolecular interactions.

Density functional theory (DFT) and time-dependent DFT (TDDFT) calculations further support this interpretation. Geometry optimizations initiated from the crystallographic structures indicate that near-cofacial dimer arrangements preferentially stabilize the excited state through enhanced intermolecular orbital overlap, whereas slipped or displaced geometries reduced electronic coupling and lead to weakly emissive, nonexcimer states. Thus, the calculations provide an electronic-structure basis for the experimentally observed dependence of the emission properties on crystal packing.

To further analyze molecular packing effects, layer-by-layer crystal structures were examined using X-ray diffraction data, Figure 3. The *tert*-butyl substituted derivatives display increased intermolecular separations (Table 3) with steric hindrance, which limits effective π···π interactions between the fluorene cores. These systems form discrete dimeric motifs stabilized by weak π-stacking and C–H···π contacts, resulting in reduced electronic coupling. In contrast, fluorene (and, by analogy, 2,7-dmF based on its photophysical behavior) exhibits dense molecular packing that promotes efficient intermolecular electronic coupling and excimer formation.

The structural requirements identified here are consistent with previous gas-phase studies by Saigusa and Itoh, who observed excimer fluorescence from fluorene van der Waals dimers under collision-free conditions in a supersonic jet.^56^ Excitation of these dimers produced characteristic excimer fluorescence centered at ∼360 ± 10 nm, demonstrating that sufficiently close, near-cofacial π–π interactions are sufficient to stabilize emissive excimer states in the absence of crystal packing, supporting the mechanistic interpretation proposed for the present solid-state systems.

To quantitatively correlate structure and electronic coupling, dimer geometries were optimized in both the ground and first singlet excited states using crystallographic coordinates as initial structures. All calculations were performed considering gas phase conditions. Interplanar and intermolecular distances are summarized in Figure 3 and Table 3.

For fluorene, optimized dimers retain relatively short intermolecular distance (average ∼ 4.49 Å), consistent with strong coupling. In contrast, *tert*-butyl-substituted derivatives exhibit significantly larger separations and geometric displacements, limiting effective orbital overlap. In the crystal of 2,7-*dtb*F and 3,6-*dtb*F, centroid-to-centroid distances between interacting fluorene units are 9.121 Å and 4.018 Å, respectively, corresponding to shifts of 0.054 Å and 0.213 Å relative to the experimental crystal structures.

Overall, the combined crystallographic, photophysical, and computational results establish a clear structure–property relationship linking steric substitution, molecular packing, intermolecular electronic coupling, and excited-state relaxation. Efficient excimer emission is therefore favored by near-cofacial π-stacked arrangements that maximize intermolecular orbital overlap, whereas sterically induced packing distortions shift the excited-state dynamics toward weakly coupled or monomer-like emissive regimes. These findings demonstrate that steric engineering provides an effective strategy for modulating intermolecular electronic coupling and, consequently, the balance between localized and excimer-like excited states in fluorene-based molecular solids.

### DFT/TDDFT Analysis of Ground and Excited State Structures and Electronic Transitions

To gain deeper insight into the fluorescence mechanisms and excimer formation, DFT and TDDFT calculations were performed on monomeric and dimeric species of fluorene and its derivatives. For fluorene, 2,7-*dtb*F, and 3,6-*dtb*F, dimer geometries were derived directly from experimentally determined crystal packing motifs (Figure 3), and all structures were optimized at the DFT//LC-BPBE (ω = 0.2) level, with frequency analyses confirming all stationary points as true minima on the potential energy surface.

Calculated UV–vis spectra reproduced the experimentally observed π–π* absorption bands (262–271 nm in THF, Table S6). To provide a more comprehensive comparison with the experimental spectra, the calculated vertical excitation energies for the first four singlet excited states, together with their oscillator strengths and dominant electronic configurations, are summarized in Table S6. The lowest-energy absorption band is predominantly assigned to the HOMO → LUMO transition, whereas the higher-energy bands arise from contributions of HOMO → LUMO + 1, HOMO −1 → LUMO, and HOMO −2 → LUMO excitations, depending on the substitution pattern.

Frontier molecular orbital analysis shows that both the HOMO and LUMO are localized on the fluorene core, indicating minimal electronic perturbation from alkyl substitution (Figure S8). Natural transition orbital (NTO) analysis further confirms that the S_1_ state is fluorene-centered, consistent with the experimentally observed monomer-like emission in solution. These results demonstrate that substitution does not significantly alter the intrinsic electronic structure of the chromophore. Among the derivatives, 2,7-*dtb*F exhibits the highest calculated oscillator strength for the S_0_ → S_1_ transition (*f* = 1.159), in agreement with its experimentally enhanced radiative decay rate (*k*_R_), Table S2. Overall, the calculated vertical excitation and emission energies are in excellent agreement with experimental absorption and emission data (Tables S1 and S6).

The investigation of dimers was addressed using two complementary quantum chemical approaches. First, ground- and excited-state geometry optimizations were performed to identify equilibrium structures and evaluate the stability of excimer-like configurations. In a second step, the potential energy surfaces were explored along intermolecular long-axis translational and rotational coordinates in order to assess how molecular packing influences intermolecular electronic coupling and excited-state stabilization.

To investigate the origin of the distinct solid-state emission properties, dimer models derived from the crystallographic packing arrangements were examined by TDDFT calculations. While the electronic structures of the isolated monomers remain largely unaffected by alkyl substitution, significant differences emerge upon intermolecular association.

To further elucidate the origin of excimer formation, dimer arrangements were investigated as a function of intermolecular translation and rotation coordinates (Figure 4). Dimer geometries were optimized in both S_0_ and S_1_ states in the gas phase. The intermolecular distances and packing geometries shown in Figure 4 correspond to the optimized S_0_ structures, while the corresponding S_1_ geometries were used to evaluate excimer stabilization and emission properties (Table 4). Dimer calculations reveal a strong dependence of electronic structure on intermolecular geometry.

**Figure 4:**
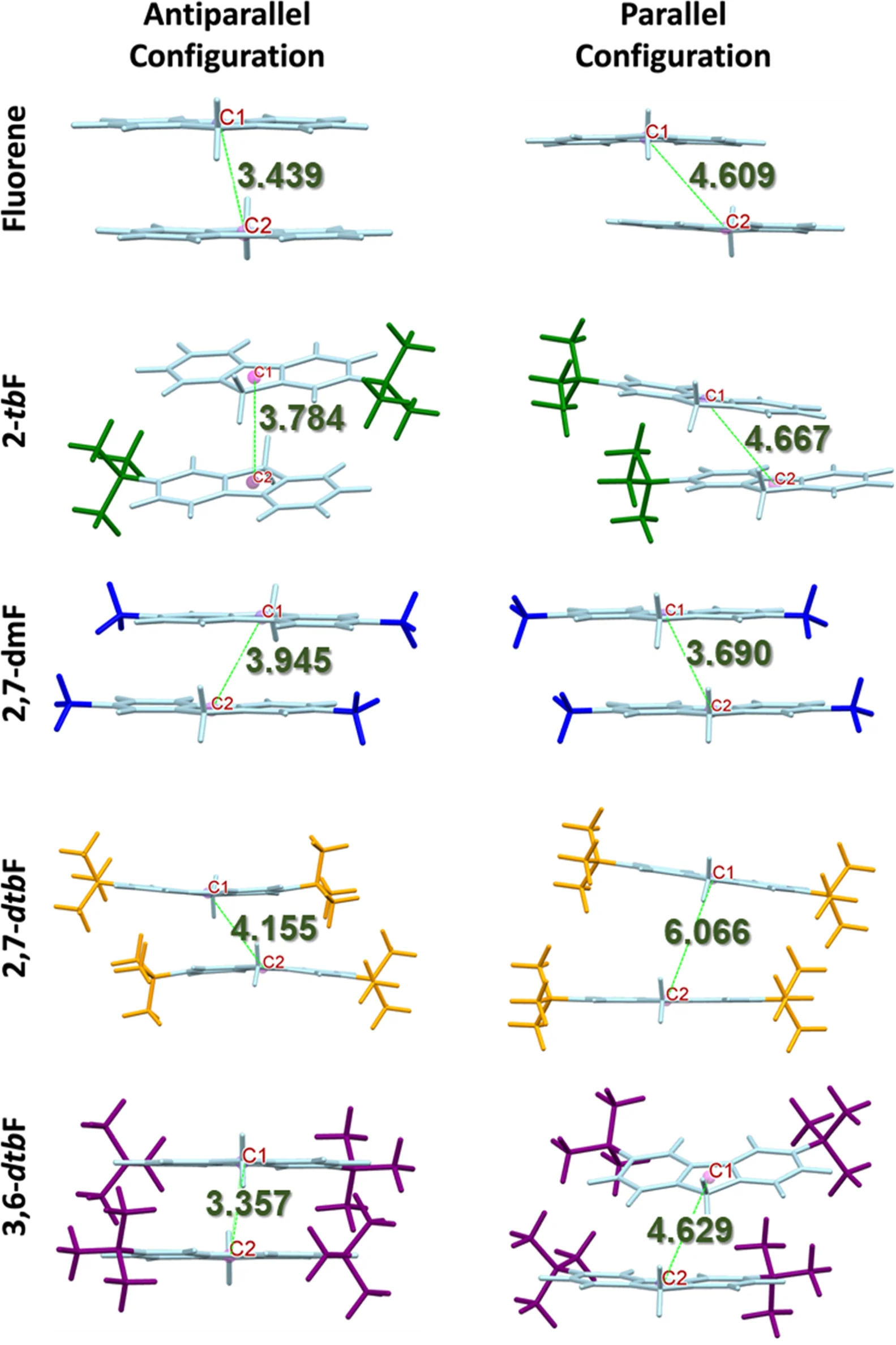
Optimized ground-state (S_0_) geometries of the antiparallel and parallel dimer configurations of fluorene and its derivatives in the gas phase. The intermolecular distances (centroid-to-centroid) between the fluorene units are indicated and summarized in Table 4.

**Table 4:** Displacement Parameters of Dimer Geometries Predicted by TDDFT Calculations in the Optimized Ground and First Singlet Excited States and Their Correlation With the Tendency for Excimer Formation

	antiparallel			parallel		
compound	S_0_ → S_1_ distance change (Å)^a^	π–π interaction	excimer tendency	S_0_ → S_1_ distance change (Å)^a^	π–π interaction	excimer tendency
fluorene	3.44 → 3.15	strong	strong	4.61 → 3.71	weak	N.P
2-*tb*F	3.89 → 3.40	weak	N.P	4.67 → 4.19	negligible	N.P
2,7-dmF	3.95 → 3.25	strong	strong	3.69 → 3.16	strong	strong
2,7-*dtb*F	4.16 → 3.76	weak	N.P	6.07→ 5.81	negligible	N.P
3,6-*dtb*F	3.36 → 3.14	strong	strong	4.63→ 3.95	weak	N.P

a Intermolecular distances between the center of mass of the molecules. N.P.: Not predicted.

In all systems, dimer formation stabilizes the lowest excited state, leading to pronounced bathochromic shifts relative to the corresponding monomers. This effect is particularly evident for 2,7-dmF, where the calculated excitation wavelength increases from 296 nm for the monomer to 387 nm for the parallel dimer geometry.

Despite these large red shifts, the lowest-energy dimer excitations exhibit dramatically reduced oscillator strengths compared to the corresponding monomer transitions. For parallel and antiparallel arrangements, oscillator strengths become nearly zero, whereas perpendicular geometries retain only weakly allowed transitions. NTO analysis indicates that these excitations remain dominated by HOMO → LUMO character, suggesting that dimerization does not fundamentally change the underlying electronic transition. Instead, aggregation primarily modifies the intermolecular electronic coupling, resulting in a redistribution of the transition dipole moment and the formation of low-energy states with weak optical activity.

These calculated trends provide a molecular-level rationale for the packing-dependent photophysical behavior observed experimentally. Cofacial π–π arrangements promote stronger intermolecular coupling and excited-state stabilization, producing low-energy excited states characterized by substantial red shifts and strongly reduced oscillator strengths. Such electronic signatures are consistent with the formation of excimer-like excited states and support the experimentally observed emergence of excimer emission in densely packed molecular assemblies. Conversely, geometries with reduced orbital overlap display weaker electronic coupling and partial recovery of transition intensity, in agreement with the suppression of excimer formation observed for sterically hindered derivatives.

In cofacial antiparallel configurations, as found in fluorene, both HOMO and LUMO become delocalized over the two monomer units, indicating strong intermolecular electronic coupling. In contrast, for 2,7-*dtb*F in the parallel configuration, orbitals remain localized on individual monomers, reflecting electronic decoupling induced by steric hindrance. Consistent with experiment, dimerization lowers the excitation energy relative to the monomer, accounting for the red-shifted absorption observed in condensed phases. The degree of this stabilization correlates directly with the extent of π–π overlap imposed by molecular packing.

Analysis of dimer geometries as a function of intermolecular separation reveals a continuous evolution of excited-state character. At large separations (>8 Å), the S_1_ state is localized and purely locally excited (LE). As the monomers approach, increasing intermolecular orbital overlap promotes the development of an intermolecular charge-transfer (CT) component, arising from partial electron-density transfer between adjacent fluorene units. At short intermolecular separations (≈3.2–3.5 Å), the excited state evolves into a hybrid local and charge-transfer (HLCT) state, in which the wave function combines locally excited (LE) and intermolecular CT character. This mixed LE/CT nature is consistent with the electronic structure expected for stabilized excimer states.

Upon excitation, excimer-forming systems undergo significant structural relaxation toward more cofacial-like geometries, accompanied by contraction of intermolecular distances and enhanced π–π overlap (Table 4). In this regime, the S_1_ state becomes significantly delocalized across both monomer units, stabilizing the excimer and producing the characteristic red-shifted emission. The calculated oscillator strength (*f*) of this state approaches zero, reflecting the highly symmetric and delocalized nature of the excimer state. In cofacial dimer geometries, the transition dipole moment associated with the S_1_ → S_0_ transition is strongly reduced owing to the symmetry of the excited-state wave function, resulting in a weakly allowed transition and very low oscillator strengths, as commonly observed for excimer emission.^57,58^

Importantly, the enhanced emission observed in the most efficient dimeric arrangements can be rationalized in terms of increased structural rigidity imposed by the sterically directed packing motifs. In these configurations, the excimer-forming geometry corresponds to a relatively deep and well-defined minimum on the excited-state potential energy surface, where large-amplitude structural relaxation is significantly restricted. This conformational confinement suppresses nonradiative decay pathways associated with extensive nuclear reorganization, thereby increasing the relative contribution of radiative decay pathways. As a result, these rigidified excimer states exhibit higher emission efficiency compared to more dynamically flexible or weakly bound excimer systems.

This analysis highlights that excimer emission efficiency is not exclusively governed by intermolecular proximity, but also critically depends on the degree of conformational freedom available in the excited state.

In fluorene and 2,7-dmF, this evolution leads to stable excimer states, in agreement with experimental observations. At intermediate separations (d ≈ 3.6 Å), NTOs reveal delocalization across both monomers while retaining predominantly LE character. Further compression (*d* ≈ 3.2 Å) leads to enhanced intermolecular charge-transfer contributions and the formation of a mixed local/charge-transfer (HLCT) excimer state, significantly stabilizing S_1_ state. The calculations therefore provide evidence for excimer stabilization, whereas its emissive nature is established experimentally by the high solid-state fluorescence quantum yields and time-resolved spectroscopic measurements. In contrast, *tert*-butyl substituted derivatives maintain larger intermolecular separations (>3.8 Å), preventing sufficient orbital overlap and suppressing excimer stabilization.

Calculated emission energies for fluorene dimers (366–396 nm, depending on configuration) are significantly red-shifted relative to monomer emission and are in good agreement with the experimental values (∼363 nm), with the small discrepancy being consistent with the expected overestimation of gas-phase TDDFT calculations. A clear correlation is observed between the extend π–π overlap and the emission energy, with increased cofacial stacking leading to stronger delocalization and larger bathochromic shifts (Figure 5).

**Figure 5:**
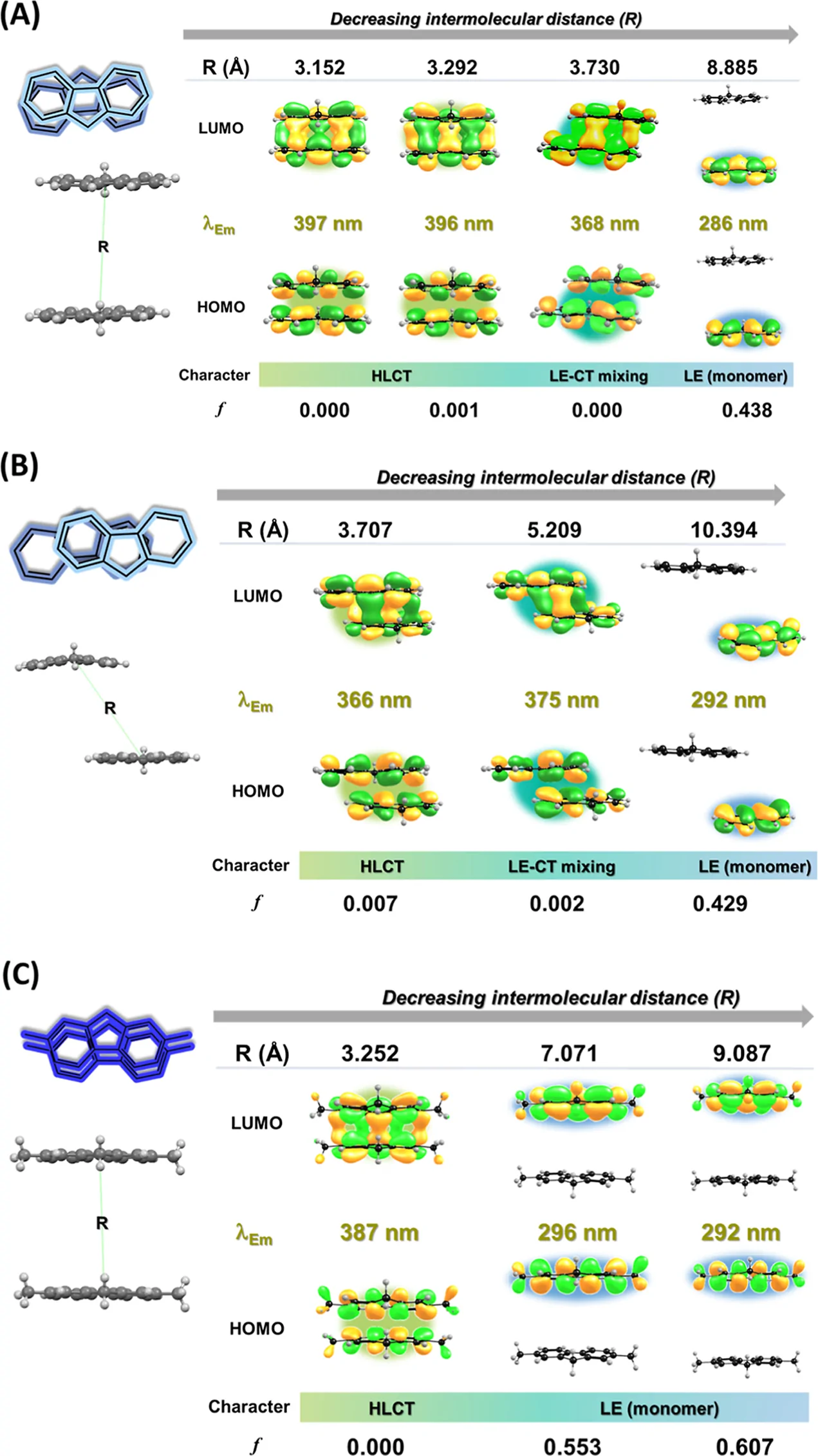

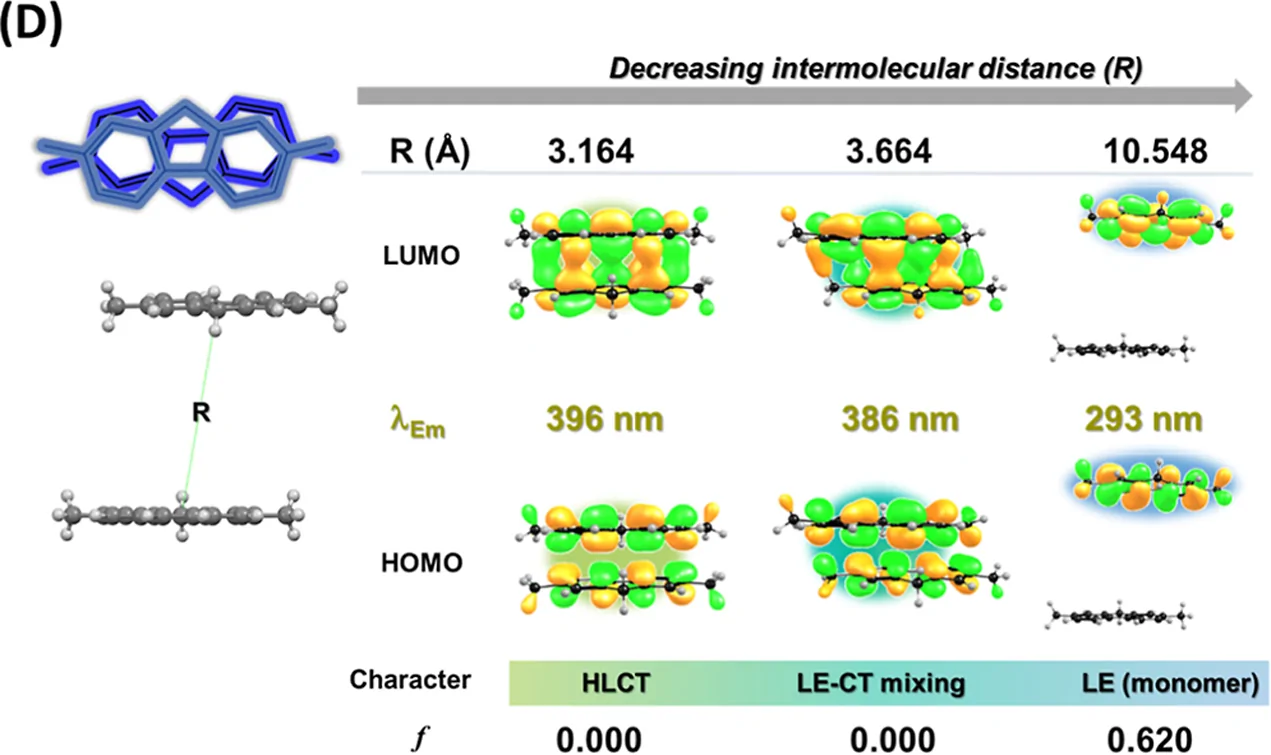
Evolution of the electronic structure as a function of intermolecular separation in fluorene and 2,7-dmF dimers upon excitation. (A) Fluorene dimer in antiparallel configuration; (B) fluorene dimer in parallel configuration; (C) 2,7-dmF dimer in parallel configuration; (D) 2,7-dmF dimer in antiparallel configuration. Excited state (S_1_) relaxation leads to reduced intermolecular distances and increased π–π overlap, favoring excimer formation. Natural transition orbitals (NTOs) illustrate the evolution from locally excited (LE, monomer-like) states at long intermolecular distances to states with increasing LE-CT mixing and hybrid local and charge-transfer (HLCT) character at shorter distances.

Figure 5 summarizes the evolution of the electronic structure as a function of intermolecular separation. The LE-CT mixing reflects the progressive admixture of locally excited (LE) and intermolecular charge-transfer (CT) character in the S_1_ state. At short intermolecular separations, this evolution gives rise to a hybrid local and charge-transfer (HLCT) excited state, reflecting the mixed electronic nature of the excimer.

Crystal-derived dimer models further confirm this relationship. For fluorene (crystal “B”), packing motifs with shorter intermolecular distances yield the strongest excimer emission (Figure S9). For 2,7-*dtb*F, steric congestion imposed by the *tert*-butyl groups prevents efficient cofacial stacking, resulting in weakly coupled or monomer-like emission with a calculated maximum at 324 nm (*f* = 0.547), in excellent agreement with experiment (Figure S10). For 3,6-*dtb*F, two distinct dimer motifs were identified: one with significant π–π overlap but poor agreement with experimental emission and no rise-time component, and a second motif featuring weakly coupled fluorene units that accurately reproduces the observed solid-state spectrum (Figure S11 and Table S7).

While these calculations demonstrate the importance of intermolecular separation for excimer stabilization, the relative orientation of the fluorene units is also expected to influence the stability of the dimer. To evaluate this effect, constrained rotational energy scans were performed for representative fluorene and 2,7-dmF dimers. In each case, one fluorene unit was rotated while maintaining the intermolecular separation fixed at 3.2 Å, corresponding to the characteristic π-stacking distance identified above. This approach isolates the effect of the relative molecular orientation independently of intermolecular separation, allowing the influence of steric interactions on the rotational energy profile to be directly evaluated.

The calculated potential energy surfaces (Figure S12) reveal well-defined rotational minima for both dimers, although the preferred mutual orientation depends strongly on substitution. The fluorene dimer exhibits a global minimum at a rotation angle of approximately 18°, whereas the preferred orientation shifts to approximately 156° for the 2,7-dmF dimer. Furthermore, the rotational barrier increases from approximately 9.4 kcal mol^–1^ for fluorene to approximately 12.8 kcal mol^–1^ for 2,7-dmF, indicating that methyl substitution substantially modifies the rotational energy profile and energetically favors a more specific mutual arrangement of the fluorene units.

In contrast, the calculated vertical excitation energies vary only slightly throughout the rotational scans, despite the pronounced differences in the corresponding potential energy surfaces. These results suggest that the experimentally observed enhancement of excimer emission is not primarily associated with significant changes in the excitation energy itself, but rather with the stabilization of preferred dimer geometries imposed by steric effects. Although the present calculations do not explicitly describe excited-state dynamics or radiative decay processes, they provide a plausible structural basis for the enhanced excimer emission observed experimentally.

Taken together, the distance-dependent excited-state calculations, the rotational energy scans, and the crystal-derived dimer models establish a direct mechanistic link between molecular packing, intermolecular electronic coupling, and excited-state character. Excimer formation emerges from a continuous evolution of the excited state with decreasing intermolecular separation, while steric substitution further modulates the rotational energy profile, defining energetically preferred dimer geometries. The combined effects of intermolecular distance and relative molecular orientation govern the extent of electronic coupling and determine whether efficient excimer emission is enabled or suppressed.

### Tuning Excimer Emission in Fluorene-Diamantane Mixtures: A Packing-Control Strategy

Diamantane, a rigid and sterically bulky hydrocarbon, was employed as an inert diluent to modulate intermolecular packing without perturbing the electronic structure of fluorene. Physical mixtures prepared by mechanical grinding enable independent control of intermolecular separation, providing a noncovalent strategy to regulate excimer formation through steric effects.

As shown in Figure 6, the emission profile evolves systematically with increasing diamantane content, establishing a direct correlation between molecular packing and photophysical behavior. All mixtures exhibit vibronically structured emission at 337–344 nm, assigned to fluorene monomer emission. In contrast, the broad excimer band centered at ∼360 nm progressively decreases in intensity and is fully suppressed at high diamantane fractions. This behavior demonstrates that increasing intermolecular separation disrupts cofacial π–π interactions required for excimer stabilization.

**Figure 6:**
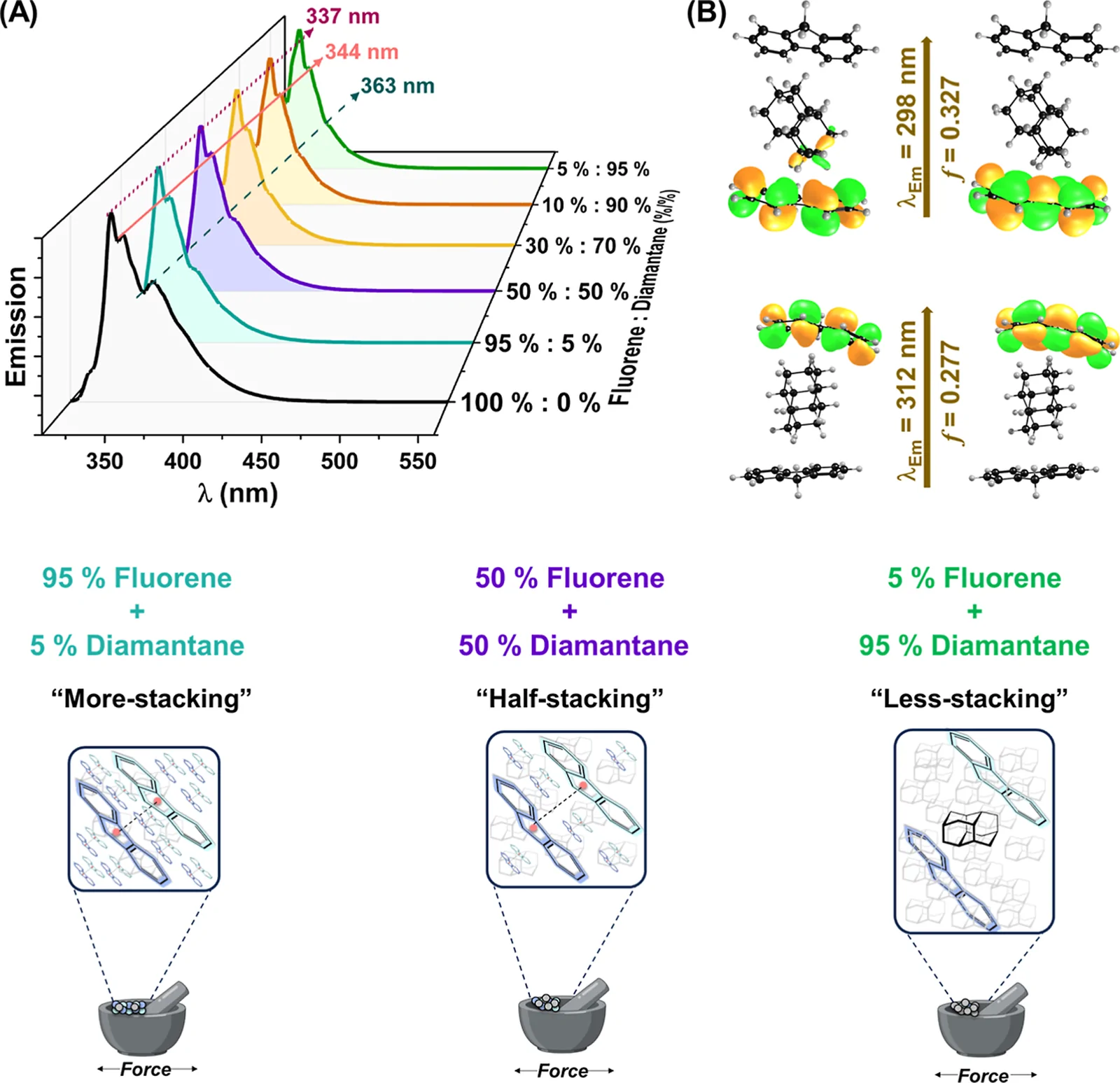
(A) Emission spectra of fluorene: diamantane mixtures showing reduced excimer emission (∼363 nm) with increasing diamantane content. Diamantane acts as a steric diluent, limiting π–π stacking and favoring monomer emission (337–344 nm). (B) Optimized geometry and frontier molecular orbitals of a fluorene dimer perturbed by a diamantane unit. The bulky diamantane moiety induces significant steric hindrance, increasing the centroid-to-centroid distance between fluorene units and disrupting cofacial π–π stacking.

Time-resolved fluorescence measurements provide direct kinetic evidence for this packing-controlled mechanism. Upon excitation at 282 nm and monitoring the excimer-dominated emission region, pristine fluorene exhibits a pronounced rise-time component (∼8 ns in a 95% fluorene: 5% diamantane mixture), followed by biexponential decay with lifetimes of 19 and 37 ns, Figures S13 and S14. The presence of a rise time confirms that excimer emission arises from excited-state conversion from initially excited, weakly coupled configurations to the emissive excimer state (lattice-mediated structural/electronic relaxation), rather than solely from direct excitation of preassociated dimers. It is worth noting that rise components depend on both packing and the spectral window monitored; therefore, rise times are compared only within the same sample type and detection wavelength range. The long decay components are consistent with radiative relaxation from a geometrically constrained excited-state dimer.

With increasing diamantane content, both the rise-time component and long-lived excimer decay are progressively suppressed. At high dilution (>95% diamantane), excimer-associated kinetics are no longer detectable, and the emission is dominated by monomer-like decay. These results demonstrate that diamantane acts as an effective steric spacer, increasing intermolecular separation beyond the threshold required for excimer formation, schematically illustrated in Figure 6.

A similar trend is observed for 2,7-dmF, which also exhibits excimer emission in the pristine solid state. Upon incorporation of diamantane, the excimer spectral contribution decreases, while the monomer emission becomes dominant (Figures 7 and S15). Consistent with the steady-state results, time-resolved measurements confirm the disappearance of the excimer-associated rise-time component at high diamantane loadings (>95%), with exclusively monomer-like dynamics remaining (Table S8). These findings confirm that diamantane effectively disrupts the molecular proximity required for excimer formation, irrespective of the fluorene substitution pattern.

**Figure 7:**
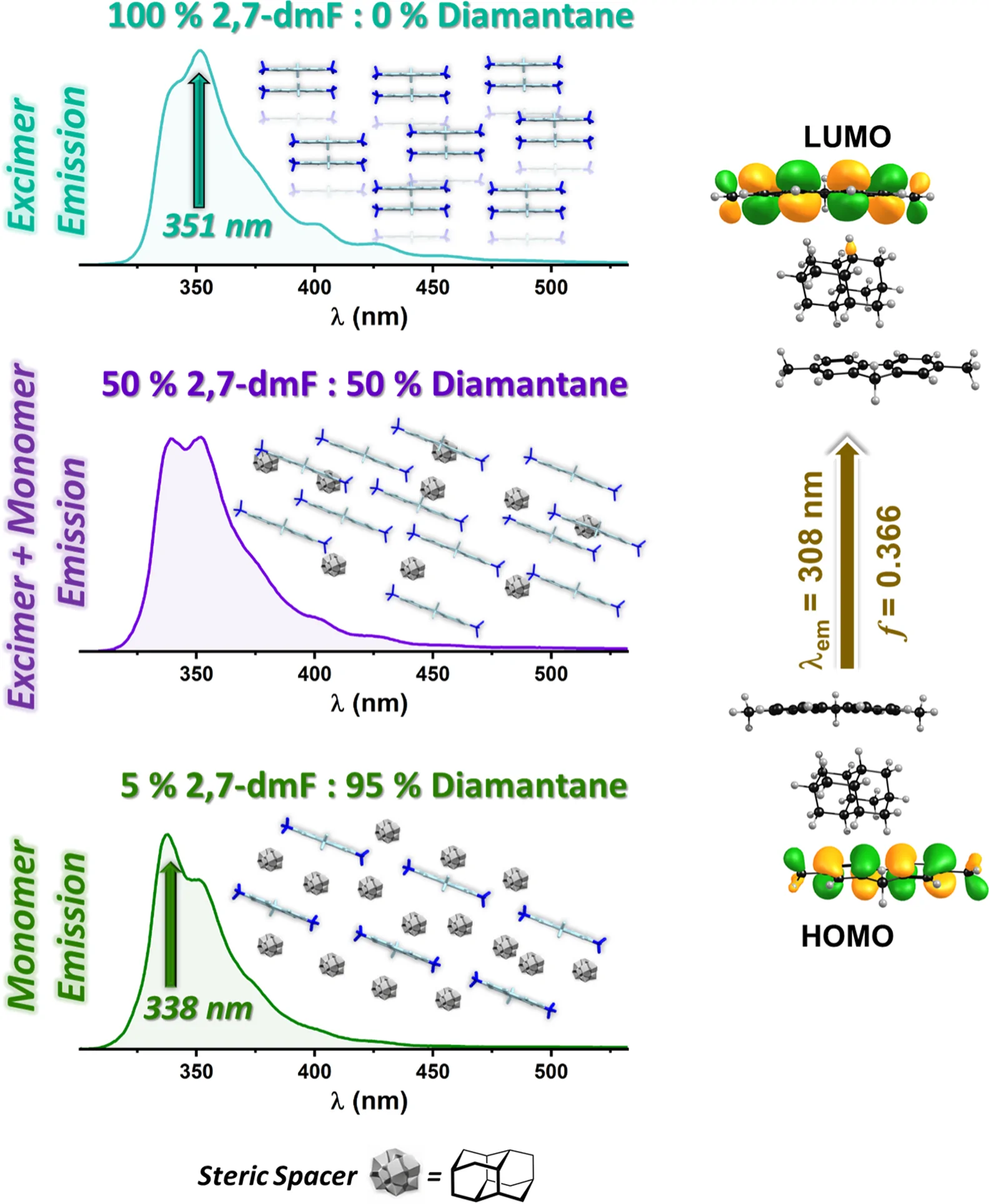
(Left panel) Experimental steady-state photoluminescence spectra of 2,7-dmF at different concentrations in a diamante-based host matrix. At high concentration (100%), the emission is slightly red-shifted (∼351 nm) and consistent with excimer formation. Upon dilution (50% and 50%), the emission exhibits a hypsochromic shift, indicating suppression of intermolecular π–π interactions. At low concentration, the emission is centered at ∼338 nm, characteristic of monomer-like emission. (Right panel) DFT-optimized structure and TDDFT-calculated natural transition orbitals (NTOs) of the 2,7-dmF dimer with a diamantane spacer. In the absence of strong electronic coupling, the excited state is localized, consistent with increased intermolecular separation from ∼3.16 Å to ∼10.08 Å, yielding negligible electronic coupling between fluorene units.

Together, these results provide an orthogonal, noncovalent validation that excimer emission can be switched off by sterically enforced dilution that increases intermolecular separation and reduces π–π overlap.

The enhanced emission observed in the sterically constrained dimers can be rationalized in terms of conformational rigidification induced by the bulky diamantane spacer. This rigidification limits structural relaxation in both the ground and excited states, thereby reducing nonradiative decay channels. In contrast to conventional excimer systems characterized by dynamic structural disorder and shallow potential energy surfaces, the present systems exhibit well-defined minima dictated by steric constraints, leading to suppressed conformational freedom and enhanced radiative emission. This suggests that emission efficiency is governed not only by electronic coupling effects but also by the degree of structural rigidity of the dimeric architecture.

To gain molecular-level insight into the role of diamantane in suppressing excimer formation, TDDFT calculations were performed on fluorene and 2,7-dmF monomers, dimers, and diamantane-separated dimer assemblies. The isolated monomers exhibit strongly allowed π–π* transitions at 297 nm (fluorene, *f* = 0.421) and 298 nm (2,7-dmF, *f* = 0.689), both dominate by HOMO → LUMO excitations.

In contrast, the corresponding dimers display low-energy excited states significantly red-shifted relative to the monomers. For fluorene, these states appear between 366 and 397 nm with negligible oscillator strengths (*f* ≈ 0.00–0.01), while the corresponding 2,7-dmF dimers exhibit analogous states between 386 and 396 nm with *f* ≈ 0, indicating weakly allowed, intermolecularly coupled excitations consistent with excimer-like character.

Upon insertion of a diamantane spacer, these low-energy aggregate states are fully suppressed. The diamantane-separated assemblies recover higher-energy transitions at 298–312 nm (fluorene), Figure 6B, and ∼308 nm (2,7-dmF), Figure 7, with moderate oscillator strengths (*f* = 0.28–0.37), and dominated by a single HOMO → LUMO contribution (>84–96%), consistent with localized monomer-like excitations.

Structural analysis provides further support for this interpretation. In the pristine fluorene and 2,7-dimethylfluorene dimers, the chromophores are separated by only 3.15 to 3.71 Å, enabling significant intermolecular orbital overlap and electronic coupling. Upon insertion of a diamantane spacer, the center-to-center distances increase to approximately 10.16 and 10.08 Å, effectively eliminating direct π–π interactions between neighboring fluorene units. Consistent with this increased separation, the calculated frontier molecular orbitals become spatially localized on individual chromophores, while no appreciable electronic density is observed on the diamantane spacer.

These theoretical results directly support the experimental observations, where increasing diamantane content suppresses excimer emission and restores monomer-like fluorescence. The calculations reveal the molecular origin of this behavior: diamantane removes the low-energy intermolecularly coupled excited states required for excimer stabilization and electronically decouples neighboring chromophores.

Together, the theoretical and spectroscopic results demonstrate that diamantane acts as an electronically inert steric spacer that suppresses excimer formation by disrupting intermolecular proximity and π–π overlap without significantly perturbing the intrinsic electronic structure of the fluorene chromophores. The identical trends observed for fluorene and 2,7-dmF further confirm that this mechanism is governed primarily by molecular packing rather than by the substitution pattern of the fluorene framework.

## Conclusions

This work shows that solid-state emission in fluorene-based molecular solids is governed by intermolecular electronic coupling controlled by molecular packing. While all compounds exhibit fluorene-centered locally excited emission in solution, their solid-state behavior diverges with respect to the steric accessibility of cofacial π–π interactions.

Unsubstituted fluorene and 2,7-dimethylfluorene (2,7-dmF) adopt antiparallel π-stacked arrangements that enable efficient intermolecular coupling and excimer-like emission, evidenced by red-shifted spectra, increased lifetimes, and the presence of a rise-time component associated with dynamic excimer formation. In contrast, *tert*-butyl substitution (2-*tb*F, 2,7-*dtb*F, and 3,6-*dtb*F) disrupts cofacial packing, suppressing excimer stabilization, and yields predominantly monomer-like or weakly coupled emissive states.

These results show that solid-state emission is not an intrinsic molecular property but arises from a continuum of intermolecular coupling regimes governed by supramolecular organization. Steric substitution provides direct control over these regimes, enabling selective activation or suppression of excimer emission without altering the electronic structure of the chromophore.

This concept is further supported using diamantane as a rigid steric spacer, which tunes intermolecular separation and inhibits excimer formation in a controlled manner. Overall, these findings establish excimer emission as a packing-dependent, designable excited-state pathway and provide a framework for tuning intermolecular electronic coupling in molecular solids. In this context, steric design operates as a practical “packing handle” to bias molecular crystals toward excimer-enabled or excimer-suppressed emissive manifolds.

## Supplementary Material



## Notes

Crystallography data; Time-resolved fluorescence data collected in solution, in thin films and powders; Phosphorescence emission and Quantum chemical calculations (PDF)

## Abbreviations

2,7-dmF: 2,7-dimethylfluorene
2-*tb*F: 2-*tert*-butylfluorene
2,7-*dtb*F: 2,7-di-*tert*-butylfluorene
3,6-*dtb*F: 3,6-di-*tert*-butylfluorene
