# Supplementary material for: Steric Control of Molecular Packing Governs Excimer Emission in Fluorene-Based Molecular Solids
Source: Chem Mater. 2026 Aug 10;38(16):8380–97. doi: 10.1021/acs.chemmater.6c01251 (PMC13528581; doi:10.1021/acs.chemmater.6c01251)
Supplement: Supplementary file 1 [file cm6c01251_si_001.pdf]

Supplementary information for

Steric Control of Molecular Packing Governs Excimer Emission in Fluorene-Based Molecular Solids

*Carla Cunha,[†] José A. Paixão,[‡] and J. Sérgio Seixas de Melo[†]**

[†] CQC, Department of Chemistry, University of Coimbra, P3004-535, Coimbra, Portugal

[‡]University of Coimbra, CFisUC, Department of Physics, Rua Larga, Coimbra 3004-516, Portugal

*e-mail: sseixas@ci.uc.pt

Table of Contents

Spectroscopic properties of fluorene derivatives in different organic solvents 3

Excitation-wavelength-dependent fluorescence emission spectra of fluorene derivatives in powder 4

Time-resolved fluorescence data for fluorene derivatives in solution, thin films, and powders 5

Phosphorescence emission at 77 K for fluorene derivatives 12

Crystallographic data 13

DFT and TDDFT calculations for fluorene derivatives 16

Time-resolved fluorescence data for fluorene: diamantane mixtures 21

Time-resolved fluorescence data for 2,7-dmF: diamantane mixtures 23

Spectroscopic properties of fluorene derivatives in different organic solvents

Table S1. Spectroscopic data for fluorene derivatives in different organic solvents at T = 293 K. Absorption of wavelengths (λ_{abs}), fluorescence emission maxima (λ_{em}), and Stokes Shifts (Δ_{SS}, cm^{-1}) are reported. Solvents: cyclohexane (Cx), tetrahydrofuran (THF), methanol (MeOH), and acetonitrile (MeCN).

Compound	Solvent	λ_{abs} (nm)	λ_{em} (nm)	Δ_{SS} (cm^{-1})
**Fluorene**	Cx	261	302	5202
	THF	263	303	5020
	MeOH	261	302	5202
	MeCN	261	302	5202
**2,7-dmF**	Cx	269	313	5226
	THF	269	314	5328
	MeOH	268	313	5365
	MeCN	268	314	5466
**2-*tb*F**	Cx	269	306	4495
	THF	269	307	4601
	MeOH	267	306	4773
	MeCN	264	306	5199
**2,7-*dtb*F**	Cx	273	310	4372
	THF	274	311	4342
	MeOH	272	310	4507
	MeCN	272	310	4507
**3,6-*dtb*F**	Cx	264	307	5305
	THF	264	307	5305
	MeOH	263	306	5343
	MeCN	263	306	5343

Excitation-wavelength-dependent fluorescence emission spectra of fluorene derivatives in powder

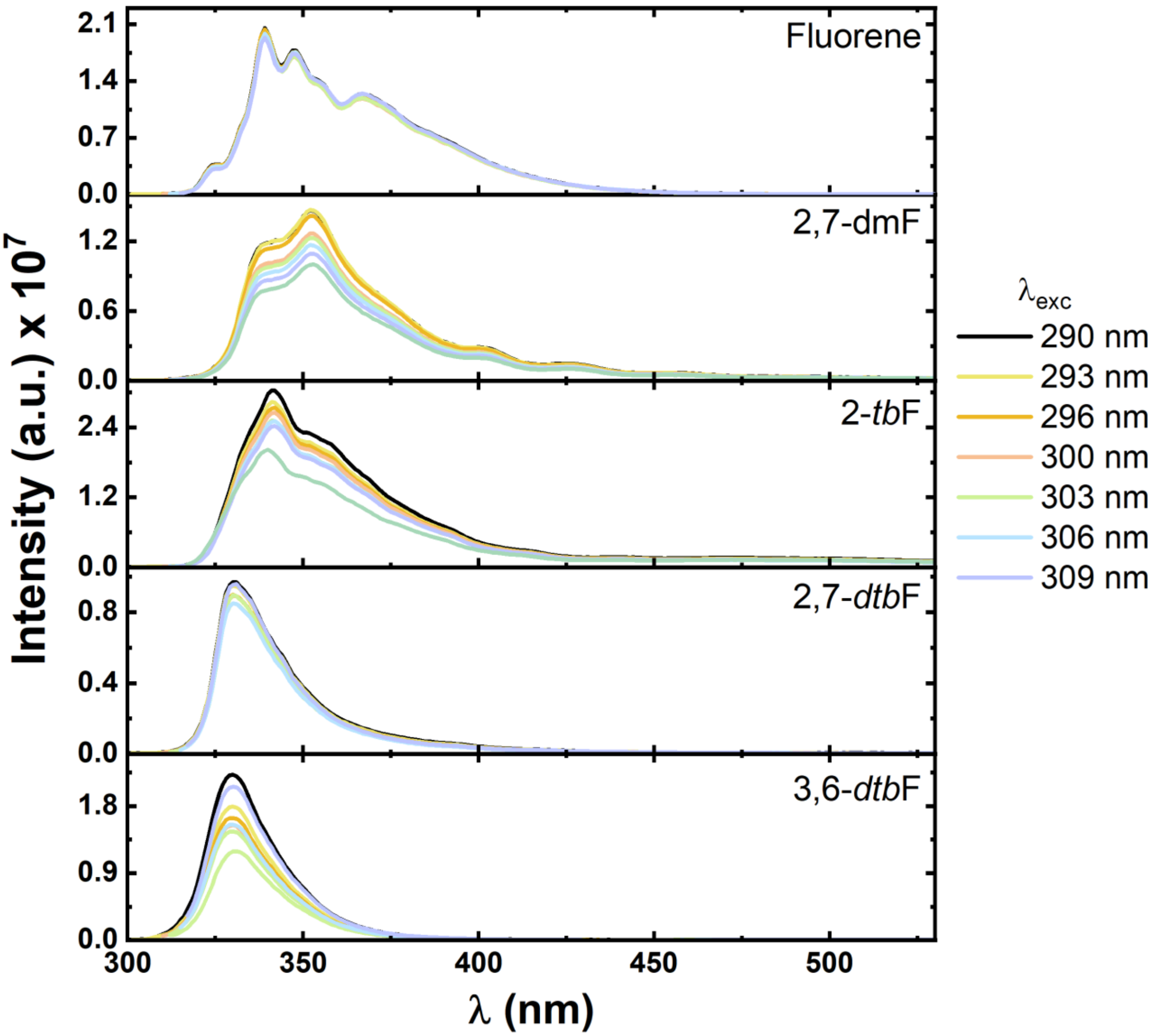

Figure S1. Excitation-wavelength-dependent fluorescence emission spectra of powder samples of fluorene, 2,7-dimethylfluorene (2,7-dmF), 2-*tert*-butylfluorene (2-*tb*F), 2,7-di-*tert*-butylfluorene (2,7-*dtb*F), and 3,6-di-*tert*-butylfluorene (3,6-*dtb*F), recorded for excitation wavelengths between 290 and 309 nm. Spectra are shown without normalization.

Time-resolved fluorescence data for fluorene derivatives in solution, thin films, and powders

To further characterize and rationalize the deactivation pathways of the first singlet excited-state, time-resolved fluorescence studies were performed in both solution and solid state (in thin films and powders). The photoluminescence (PL) intensity response with time, $I(t)$, is given by **equation 1.1**, with decay times, τ_i, and pre-exponential factors, a_i, where i stands for the number of the exponential,

$$I(t) = \sum_{i=1}^{n} a_i e^{-t/\tau_i} \qquad \textbf{(eq. 1.1)}$$

where τ_i terms are the decay times and a_i terms are the pre-exponential factors that represent the contribution of each exponential term for $t = 0$. The fractional contribution of each species [C_i (%)] was determined using the following **equation 1.2**:

$$C_i\ (\%) = \frac{a_i\ \tau_i}{\sum_{i=1}^{n} a_i\ \tau_i} \times 100 \qquad \textbf{(eq. 1.2)}$$

where n represents the number of exponential terms, a_i is the contribution of each exponential term, for $t = 0$ and τ_i is the associated decay time.

Table S2. Time-resolved fluorescence data for fluorene derivatives measured by ps/ns-TCSPC. Fluorescence lifetimes (τ_i) were obtained in aerated (O_2) and degassed (N_2 saturated) solutions, together with fluorescence quantum yields (ϕ_F) measured in different organic solvents and in the solid state (powders and thin films). Measurements were performed at excitation wavelengths λ_{exc} = 268 or 282 nm and T = 293 K. Solvents used include cyclohexane (Cx), tetrahydrofuran (THF), methanol (MeOH), and acetonitrile (MeCN).

Compound	Solvent/Medium	τ_i with O_2 (ns)	τ_i N_2 sat. (ns)	ϕ_F	$^b k_R$ (s^{-1}) x 10^8	$^b k_{NR}$ (s^{-1}) x 10^8
**Fluorene**	Cx	4.66	6.90	0.496	1.06	1.08
	THF	4.97	7.43	0.465	0.94	1.08
	MeOH	4.90	7.27	0.473	0.97	1.08
	MeCN	4.57	6.89	0.501	1.10	1.09
	Film	4.89[a]	-	0.138	0.28	1.76
	Powder	24.13[a]	-	0.655	0.27	0.14
**2,7-dmF**	Cx	4.48	6.39	0.512	1.14	1.09
	THF	4.37	6.15	0.490	1.12	1.17
	MeOH	4.44	6.66	0.487	1.10	1.16
	MeCN	4.23	6.44	0.476	1.13	1.24
	Film	3.76[a]	-	0.177	0.47	2.19
	Powder	1.58[a]	-	0.616	3.90	2.43
**2-*tb*F**	Cx	4.23	5.71	0.500	1.18	1.18
	THF	4.05	5.48	0.489	1.21	1.26
	MeOH	4.28	6.02	0.476	1.11	1.22
	MeCN	4.27	5.80	0.490	1.15	1.19
	Film	4.75[a]	-	0.083	0.18	1.93
	Powder	14.69[a]	-	0.467	0.32	0.36
**2,7-*dtb*F**	Cx	3.95	5.23	0.536	1.36	1.17
	THF	3.77	5.07	0.496	1.32	1.34
	MeOH	3.95	5.37	0.528	1.34	1.19
	MeCN	3.75	5.18	0.497	1.33	1.34
	Film	3.37[a]	-	0.100	0.30	2.67
	Powder	5.53[a]	-	0.520	0.94	0.87
**3,6-*dtb*F**	Cx	3.98	5.11	0.540	1.36	1.16
	THF	3.93	4.98	0.511	1.03	0.98
	MeOH	3.93	5.38	0.526	0.98	0.88
	MeCN	3.79	5.23	0.469	0.89	1.02
	Film	3.15[a]	-	0.091	0.29	2.89
	Powder	4.54[a]	-	0.070	0.15	2.05

[a]The decay time considered here is the major contribution component in the fluorescence decays. [b]$k_F = \frac{\phi_F}{\tau_F}$; $k_{NR} = \frac{1-\phi_F}{\tau_F}$.

Table S3. Time-resolved fluorescence data for fluorene derivatives measured by ps/ns-TCSPC. Fluorescence lifetimes (τ_i) and corresponding pre-exponential factors (a_{ij}) were obtained in solution, thin films, and powder samples. Measurements were performed at excitation wavelengths λ_{exc} = 268 or 282 nm and T = 293 K.

Compound	Medium	λ_{em} (nm)	τ_1 (ns)	τ_2 (ns)	τ_3 (ns)	a_1 (%C_1)[a]	a_2 (%C_2)[a]	a_3 (%C_3)[a]	χ^2
**Fluorene**	**Solution**	320	-	-	4.66	-	-	1.00 (100)	0.89
	**Film**	320	-	1.40	4.89	-	0.298 (11)	0.702 (89)	1.02
	**Powder**	350	11.95	24.13	59.1	-0.072	0.972 (93)	0.028 (7)	0.94
		380				-0.598	0.944 (88)	0.056 (12)	1.14
		420				-0.864	0.962 (91)	0.038 (9)	1.08
**2,7-dmF**	**Solution**	320	-	-	4.48	-	-	1.00 (100)	1.00
	**Film**	320	-	1.64	3.76	-	0.576 (37)	0.425 (63)	1.03
	**Powder**	350	0.84	1.58	3.57	-0.585	0.983 (96)	0.017 (4)	0.70
		380				-0.716	0.972 (95)	0.028 (5)	0.69
		420				-0.962	0.832 (69)	0.168 (31)	1.01
**2-*tb*F**	**Solution**	320	-	-	4.23	-	-	1.00 (100)	0.88
	**Film**	320	-	1.92	4.75	-	0.722 (51)	0.278 (49)	1.03
	**Powder**	350	1.58	14.69	41.25	0.753 (23)	0.237 (68)	0.010 (8)	0.84
		380				0.789 (26)	0.191 (58)	0.019 (16)	0.84
		420				0.559 (8)	0.313 (43)	0.128 (49)	1.14
**2,7-*dtb*F**	**Solution**	320	-	-	3.95	-	-	1.00 (100)	1.00
	**Film**	320	-	1.36	3.37	-	0.307 (15)	0.693 (85)	1.06
	**Powder**	350	1.84	5.53	11.94	0.270 (11)	0.712 (85)	0.018 (5)	1.14
		360				0.247 (9)	0.716 (82)	0.037 (9)	1.04
		380				0.120 (4)	0.783 (76)	0.097 (20)	1.07
**3,6-*dtb*F**	**Solution**	320	-	-	3.98	-	-	1.00 (100)	1.02
	**Film**	320	-	1.31	3.15	-	0.283 (14)	0.717 (86)	0.97
	**Powder**	355	0.21	0.98	4.54	0.475 (6)	0.232 (14)	0.293 (80)	1.06
		370				0.380 (3)	0.116 (5)	0.504 (92)	1.11

[a]Data in brackets is for the fractional emissive contribution (% C).

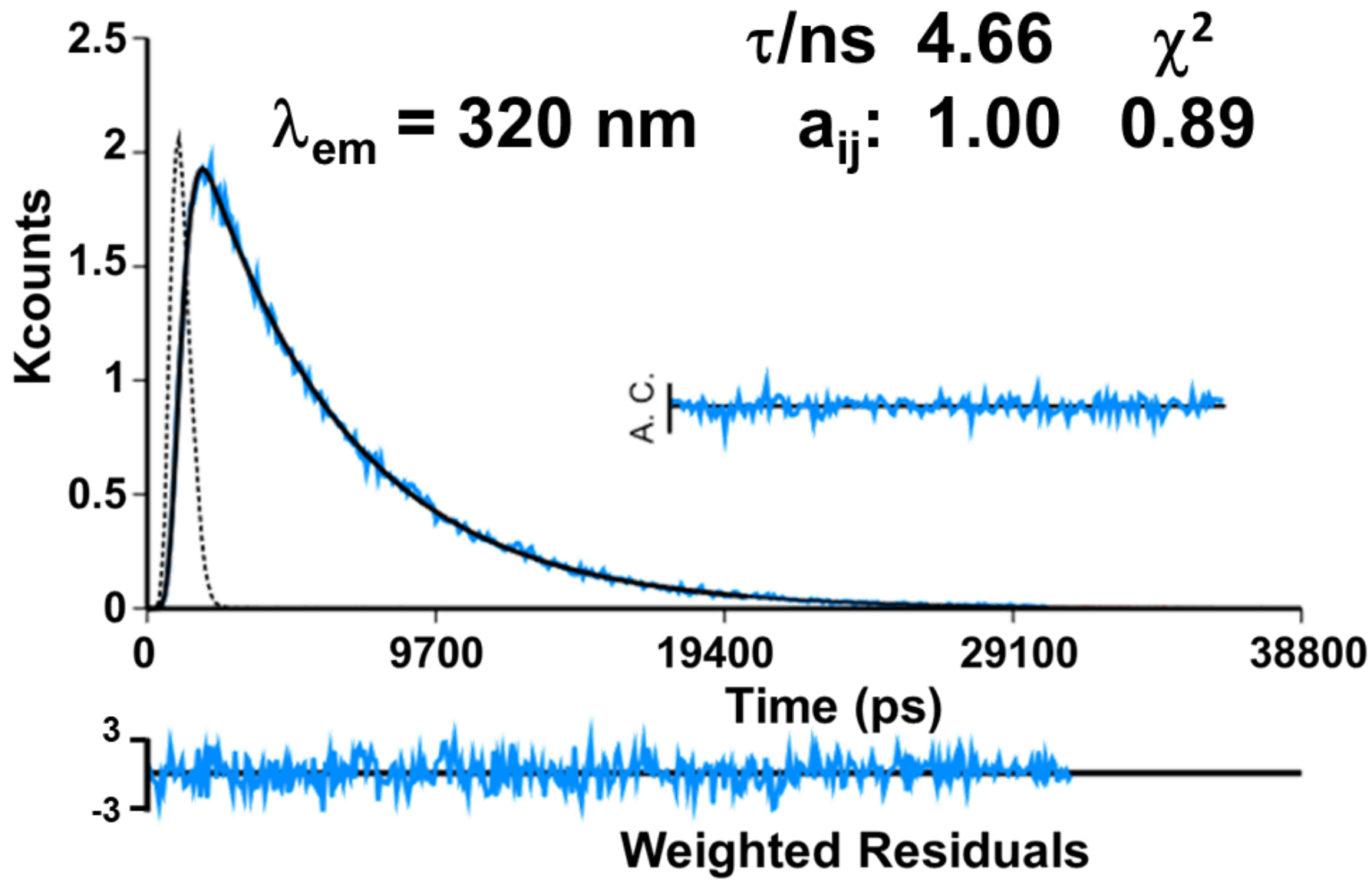

Figure S2. Room-temperature fluorescence decay of fluorene in cyclohexane (λ_{exc} = 282 nm and λ_{em} = 320 nm). The fluorescence decay times (τ_i) and the associated pre-exponential factors (a_{ij}) are presented in the inset. For a better judgment of the quality of the fit, weighted residuals, autocorrelation function (A.C.) and χ^2 values are also presented. The dashed line in the decay of corresponds to the instrumental response function.

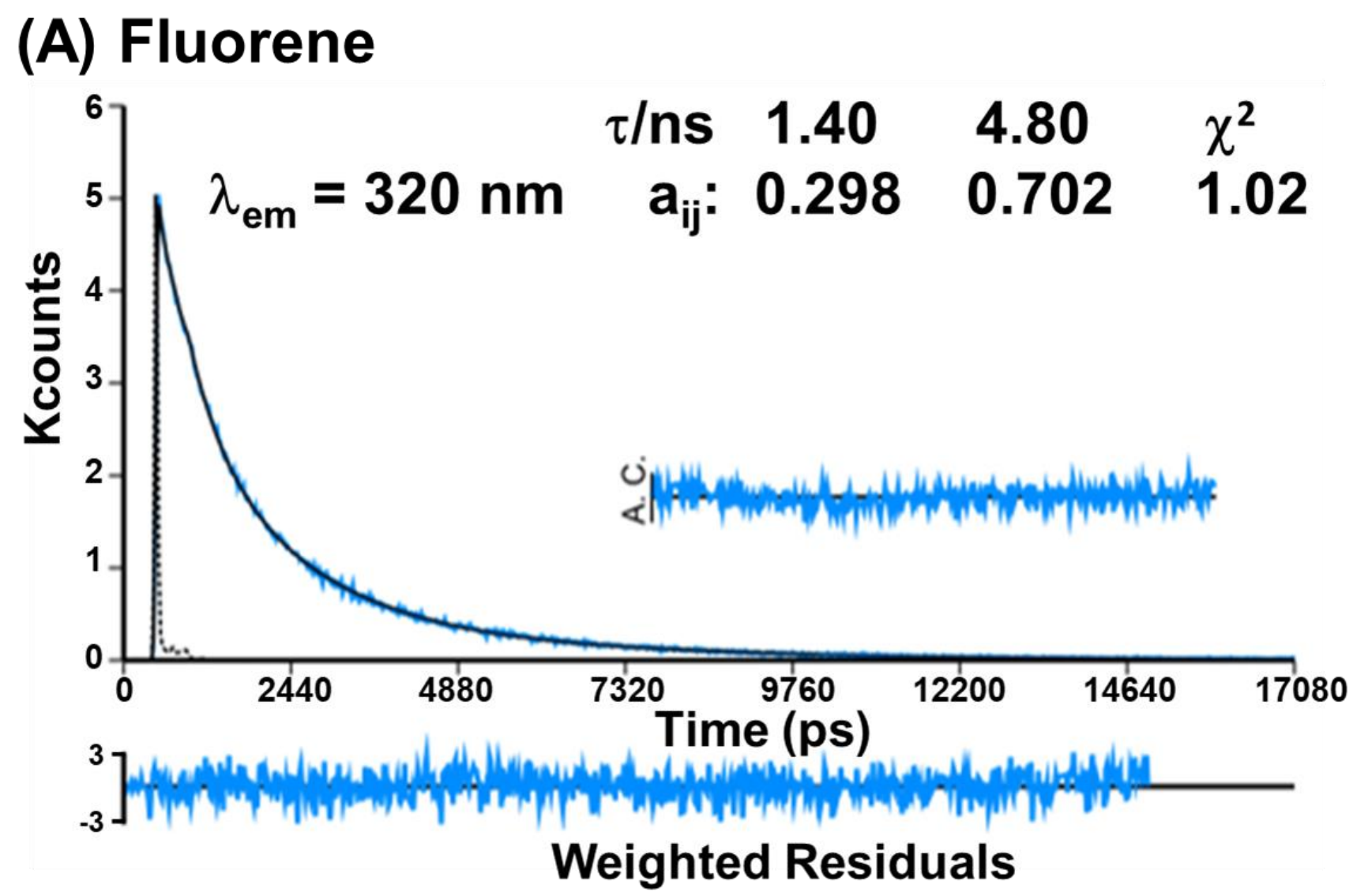

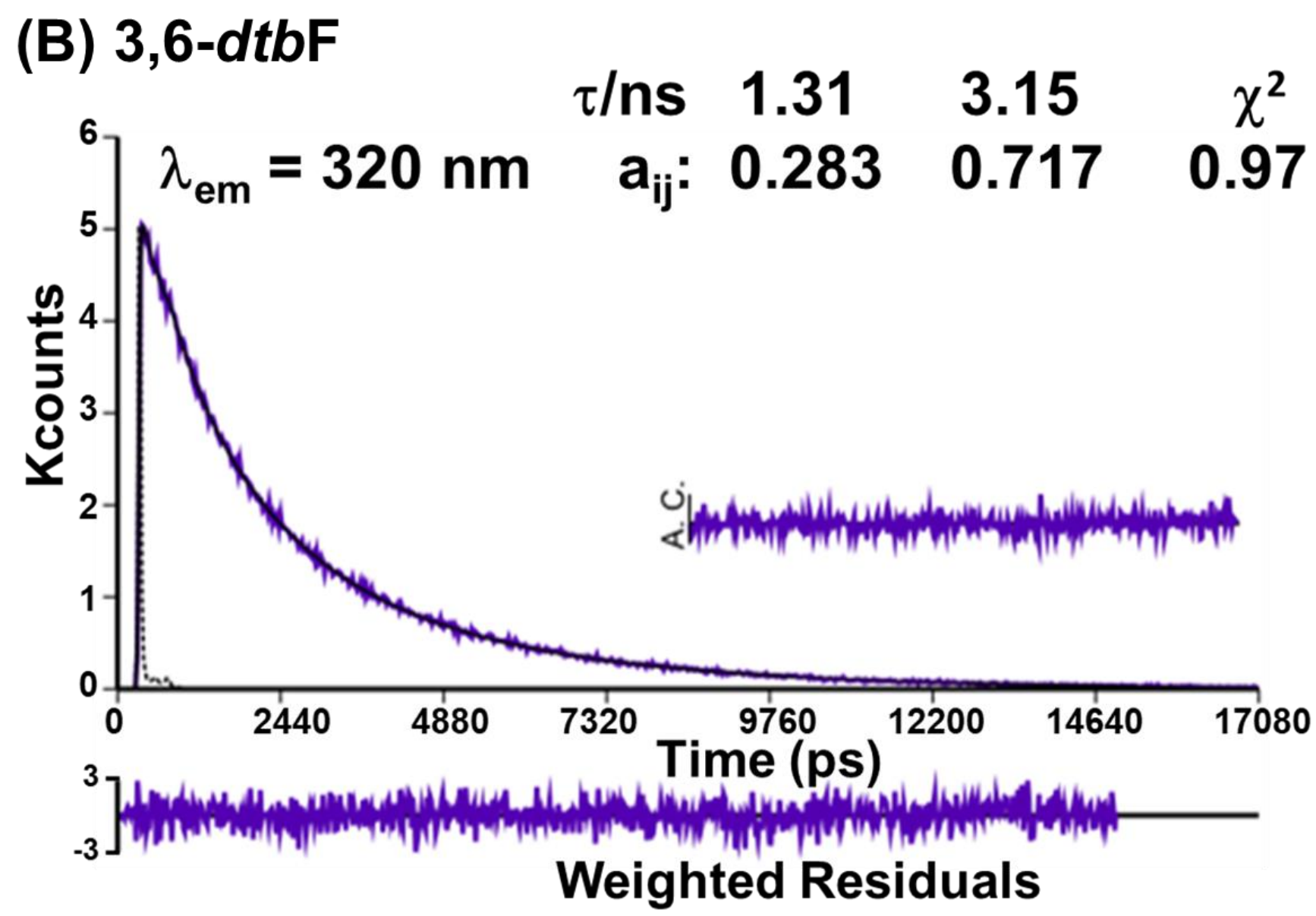

Figure S3. Room-temperature fluorescence decays of (A) fluorene and (B) 3,6-*dtb*F in thin films (λ_{exc} = 268 nm and λ_{em} = 320 nm). The fluorescence decay times (τ_{ij}) and the associated pre-exponential factors (a_{ij}) are presented in the inset. For a better judgment of the quality of the fit, weighted residuals, autocorrelation function (A.C.) and χ^2 values are also presented. The dashed line in the decay of corresponds to the instrumental response function.

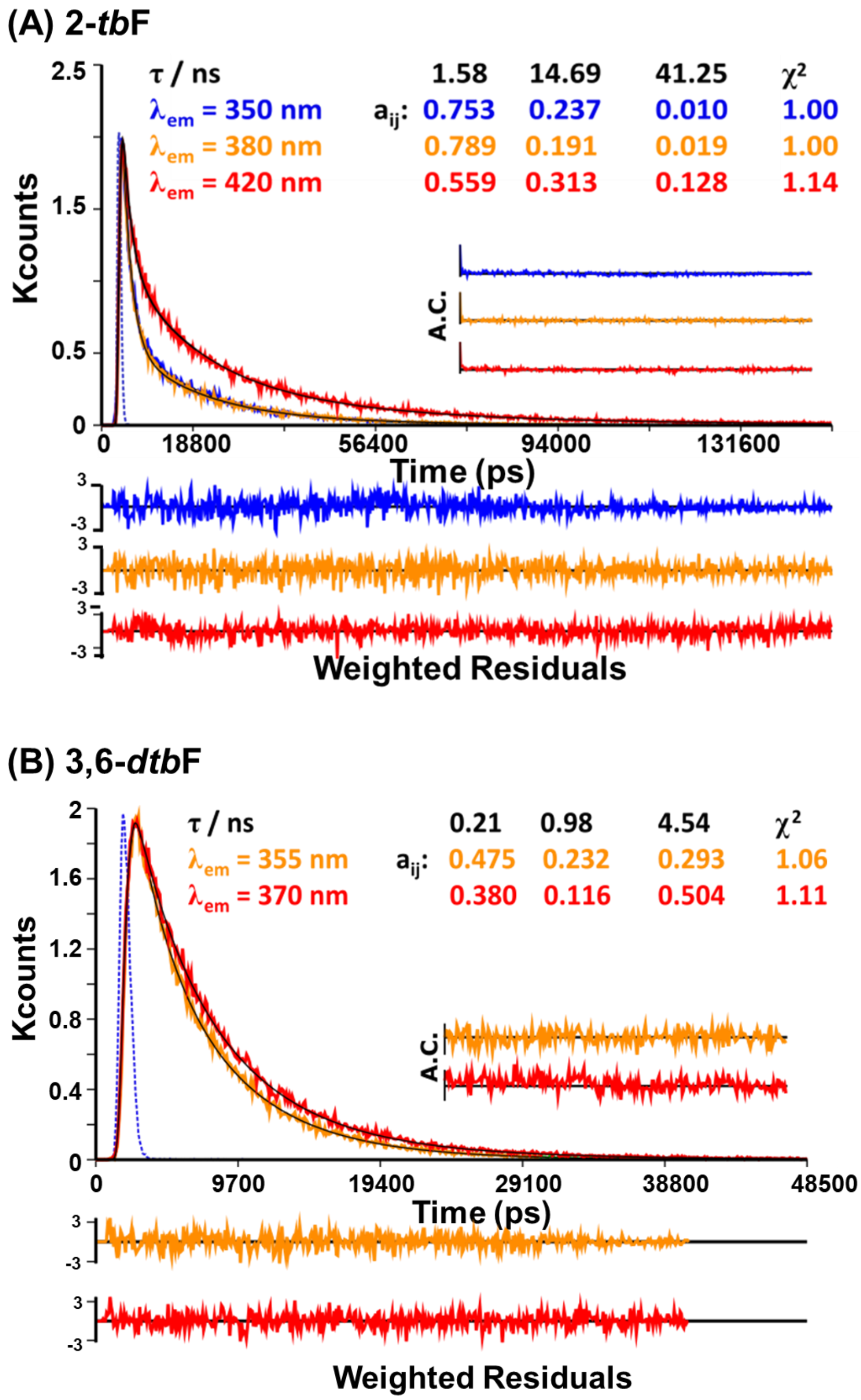

Figure S4. Room-temperature fluorescence decays of (A) 2-*tb*F and (B) 3,6-*dtb*F in powder form, recorded at λ_{exc} = 268 nm. The fluorescence decay times (τ_{ij}) and the associated pre-exponential factors (a_{ij}) are presented in the inset. For a better judgment of the quality of the fit, weighted residuals, autocorrelation function (A.C.) and χ^2 values are also presented. The dashed line in the decay of corresponds to the instrumental response function.

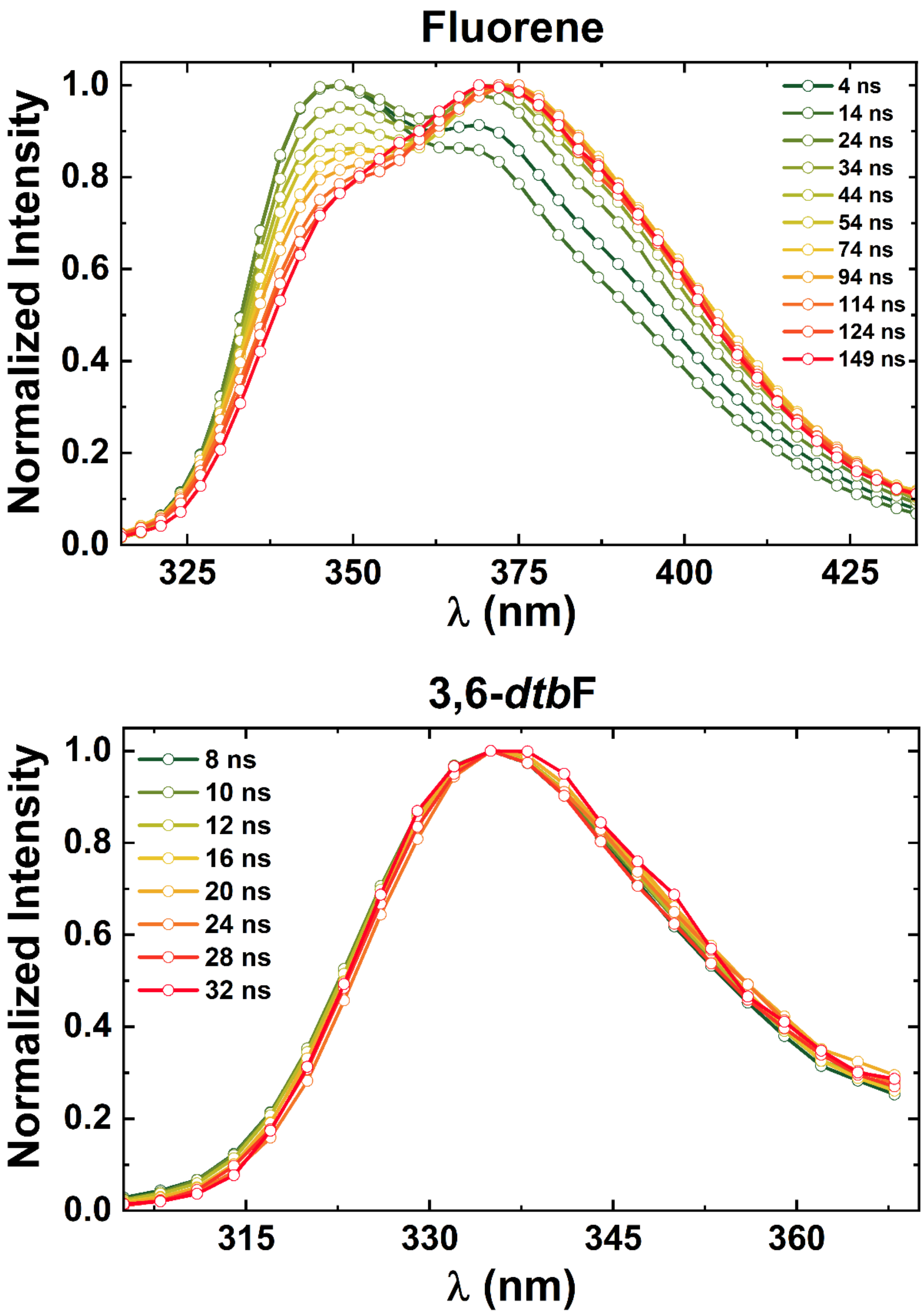

Figure S5. Normalized time-resolved emission spectra (TRES) reconstructed from wavelength-resolved TCSPC measurements by global analysis for powder samples of fluorene and 3,6-*dtb*F. Spectra recorded at representative delay times illustrate the temporal evolution of the emission profile independent of intensity changes.

Phosphorescence emission at 77 K for fluorene derivatives

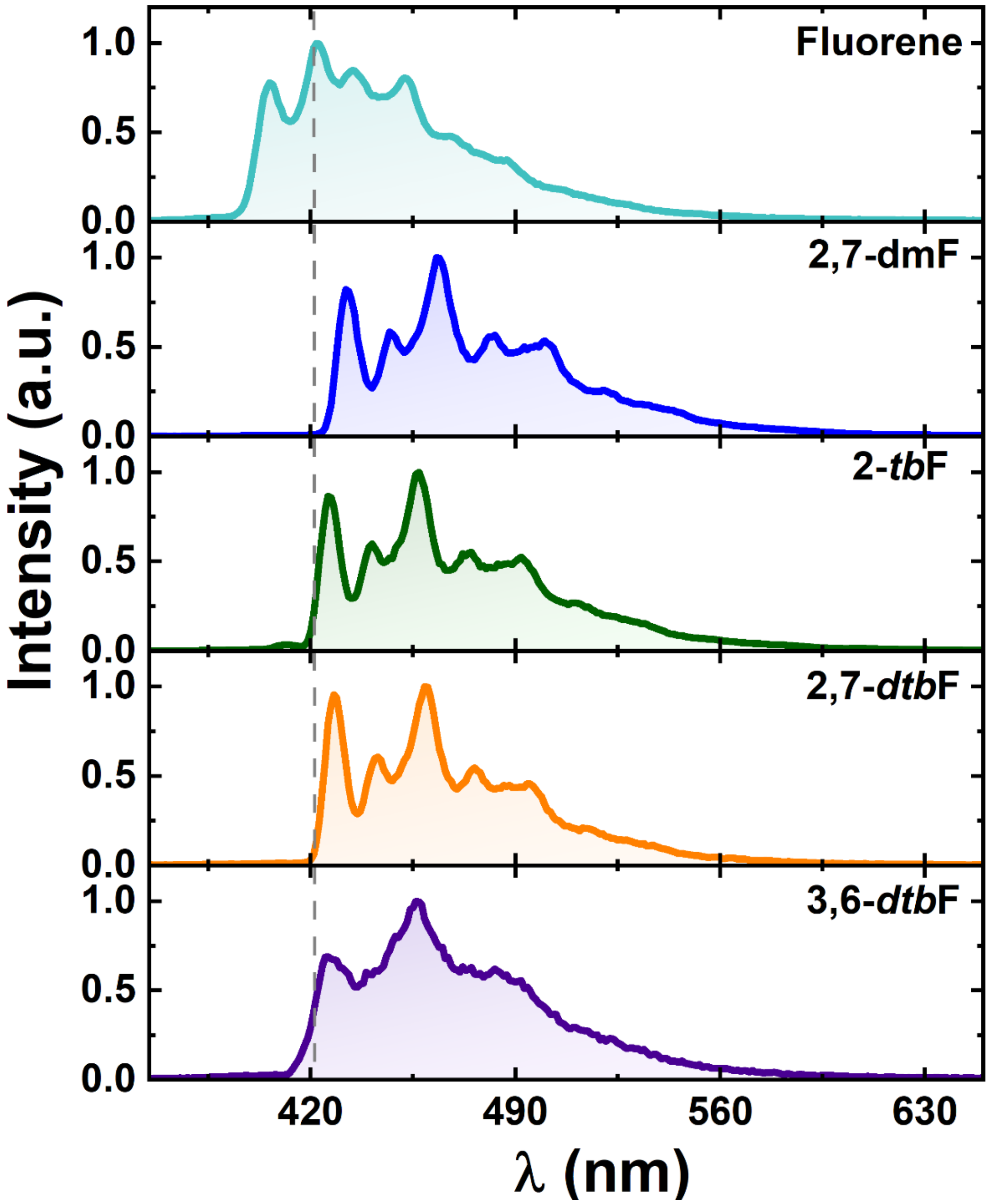

Figure S6. Normalized phosphorescence emission spectra (delay after flash, DAF = 0.5 ms) of fluorene derivatives in methlycyclohexane at T = 77 K.

Crystallographic data

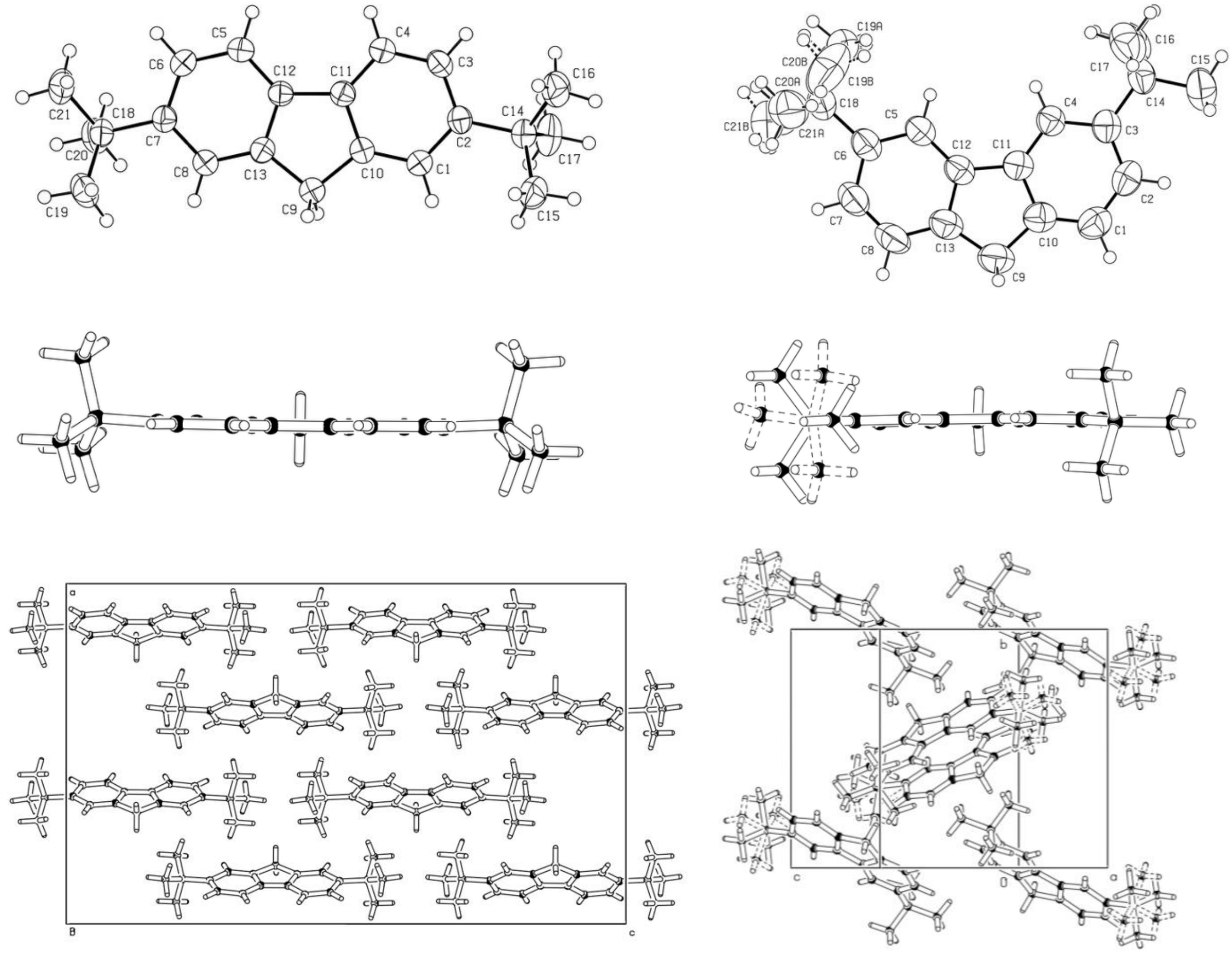

Figure S7. ORTEP plots of structures of 2,7-*dtb*F (top, left) and 3,6-*dtb*F (top, right) showing the atom numbering scheme. The anisotropic thermal displacement ellipsoids are drawn at the 50 % probability level. In the centre panels a profile view of the molecules is shown. The bottom panel depicts a view of the unit cell showing the molecular packing.

Table S4. Summary of the single-crystal X-ray data collection and crystal structure refinement for 2,7-*dtb*F.

Chemical formula (Acronym)	$C_{21}H_{26}$ (2,7-*dtb*F)
Formula weight	278.42
Color, shape	colourless/plate
Space group	*I*2/*c*
Cell volume (Å^3)	3310.80(19)
Crystal system	monoclinic
a (Å)	18.2546(7)
b (Å)	6.0438(2)
c (Å)	30.0089(8)
α (°)	90
β (°)	90.005(3)
ɣ (°)	90
Z/*Z*′	8/1
D_c (Mg m^{-3})	1.117
θ range (deg)	3.515 – 27.496
Range of *h, k, l*	–23,23;–7,7;–38,38
Reflections measured/independent	123771/7583
Reflections observed ($I > 2\sigma$)	5168
Data/restraints/parameters	7583/0/197
GOF	1.055
R_1 ($I > 2\sigma$)	0.0564
wR_2	0.1390
Function minimized	$\Sigma\, w\, (\lvert F_o\rvert^2 - S\lvert F_c\rvert^2)$
Diff. density final max/min (*e* Å^{-3})	0.226, –0.190

Table S5. Summary of the single-crystal X-ray data collection and crystal structure refinement for 3,6-*dtb*F.

Chemical formula (Acronym)	$C_{21}H_{26}$ (3,6-*dtb*F)
Formula weight	247.28
Color, shape	colourless/plate
Space group	$P2_1/c$
Cell volume (Å ^{3})	1750.69(10)
Crystal system	monoclinic
a (Å)	12.7342(4)
b (Å)	11.6793(4)
c (Å)	11.7740(4)
α (°)	90
β (°)	91.253(2)
γ (°)	90
Z/Z′	4/1
D_c (Mg m^{-3})	1.056
θ range (deg)	3.200 – 24.992
Range of *h, k, l*	–15,15;–13,13;–13,13
Reflections measured/independent	119998/3074
Reflections observed ($I > 2$ σ)	2129
Data/restraints/parameters	3074/0/228
GOF	1.048
R_1 (I >2 σ)	0.0552
wR_2	0.1498
Function minimized	$\Sigma\, w\, (\lvert F_o\rvert^2 - S\lvert F_c\rvert^2)$
Diff. density final max/min (*e* Å^{-3})	0.305, –0.154

DFT and TDDFT calculations for fluorene derivatives

Table S6. Experimental (Exp.) and theoretical (Theo.) absorption and emission maxima for fluorene derivatives in tetrahydrofuran (THF). The oscillator strengths (*f*) together with the major electronic configuration(s) are also reported. Calculations were performed at the DFT//LC-BPBE (ω=0.2) level of theory.

	$\lambda_{abs, nm}$ Exp.	$\lambda_{abs, nm}$ (*f*) Theo.	Major electronic configuration(s)		$\lambda_{em, nm}$ Exp.	$\lambda_{em, nm}$ (*f*) Theo.	Major electronic configuration(s)
Fluorene	262	262 (0.433)	$S_0 \rightarrow S_1$	HOMO → LUMO (73 %)	302	304 (0.866)	HOMO → LUMO (98 %)
		251 (0.012)	$S_0 \rightarrow S_2$	HOMO -1 → LUMO (59 %)<br>HOMO → LUMO +2 (31 %)			
		250 (0.189)	$S_0 \rightarrow S_3$	HOMO → LUMO +1 (59 %)<br>HOMO → LUMO (23 %)			
		210 (0.059)	$S_0 \rightarrow S_4$	HOMO -2 → LUMO (68 %)<br>HOMO → LUMO +1 (26 %)			
2,7-dmF	269	271 (0.607)	$S_0 \rightarrow S_1$	HOMO → LUMO (77 %)	310	315 (1.067)	HOMO → LUMO (98 %)
		259 (0.228)	$S_0 \rightarrow S_2$	HOMO → LUMO +1 (67 %)<br>HOMO → LUMO (19 %)			
		253 (0.005)	$S_0 \rightarrow S_3$	HOMO -1 → LUMO (48 %)<br>HOMO → LUMO +2 (40 %)			
		211 (0.056)	$S_0 \rightarrow S_4$	HOMO -2 → LUMO (69 %)<br>HOMO → LUMO +1 (17 %)			
2-*tb*F	269	266 (0.591)	$S_0 \rightarrow S_1$	HOMO → LUMO (76 %)	313	308 (1.020)	HOMO → LUMO (98 %)
		255 (0.189)	$S_0 \rightarrow S_2$	HOMO → LUMO +1 (58 %)<br>HOMO → LUMO (19 %)			
		252 (0.011)	$S_0 \rightarrow S_3$	HOMO -1 → LUMO (48 %)<br>HOMO → LUMO +2 (32 %)			
		211 (0.099)	$S_0 \rightarrow S_4$	HOMO -2 → LUMO (70 %)<br>HOMO → LUMO +1 (22 %)			
2,7-*dtb*F	273	270 (0.782)	$S_0 \rightarrow S_1$	HOMO → LUMO (80 %)	310	314 (1.159)	HOMO → LUMO (98 %)
		260 (0.178)	$S_0 \rightarrow S_2$	HOMO → LUMO +1 (68 %)<br>HOMO → LUMO (16 %)			
		253 (0.001)	$S_0 \rightarrow S_3$	HOMO -1 → LUMO (49 %)<br>HOMO → LUMO +2 (40 %)			
		211 (0.143)	$S_0 \rightarrow S_4$	HOMO -2 → LUMO (73 %)<br>HOMO → LUMO +1 (19 %)			
3,6-*dtb*F	265	268 (0.475)	$S_0 \rightarrow S_1$	HOMO → LUMO (75 %)	307	306 (0.880)	HOMO → LUMO (98 %)
		257 (0.025)	$S_0 \rightarrow S_2$	HOMO -1 → LUMO (63 %)<br>HOMO → LUMO +2 (25 %)			
		251 (0.176)	$S_0 \rightarrow S_3$	HOMO → LUMO +1 (55 %)<br>HOMO → LUMO (20 %)			
		216 (0.161)	$S_0 \rightarrow S_4$	HOMO -2 → LUMO (61 %)<br>HOMO → LUMO +1 (30 %)			

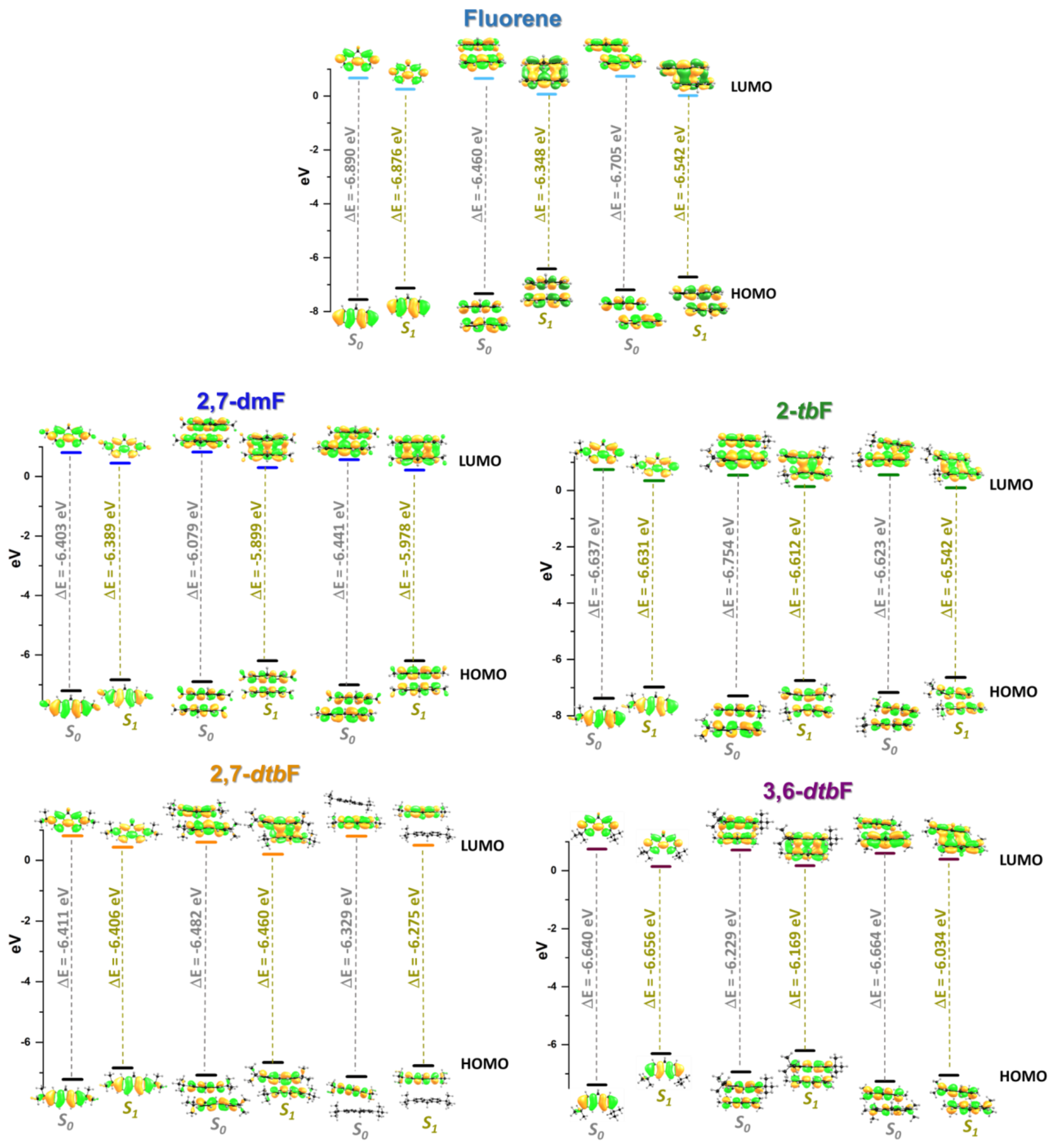

Figure S8. Molecular orbital distributions plots of HOMO and LUMO energy levels of fluorene derivatives monomer in the ground state (absorption) and fluorene derivatives dimer in the excited state (emission) predicted with DFT and TDDFT calculations, respectively. Calculations were performed at the DFT//LC-BPBE(ω=0.2) level of theory.

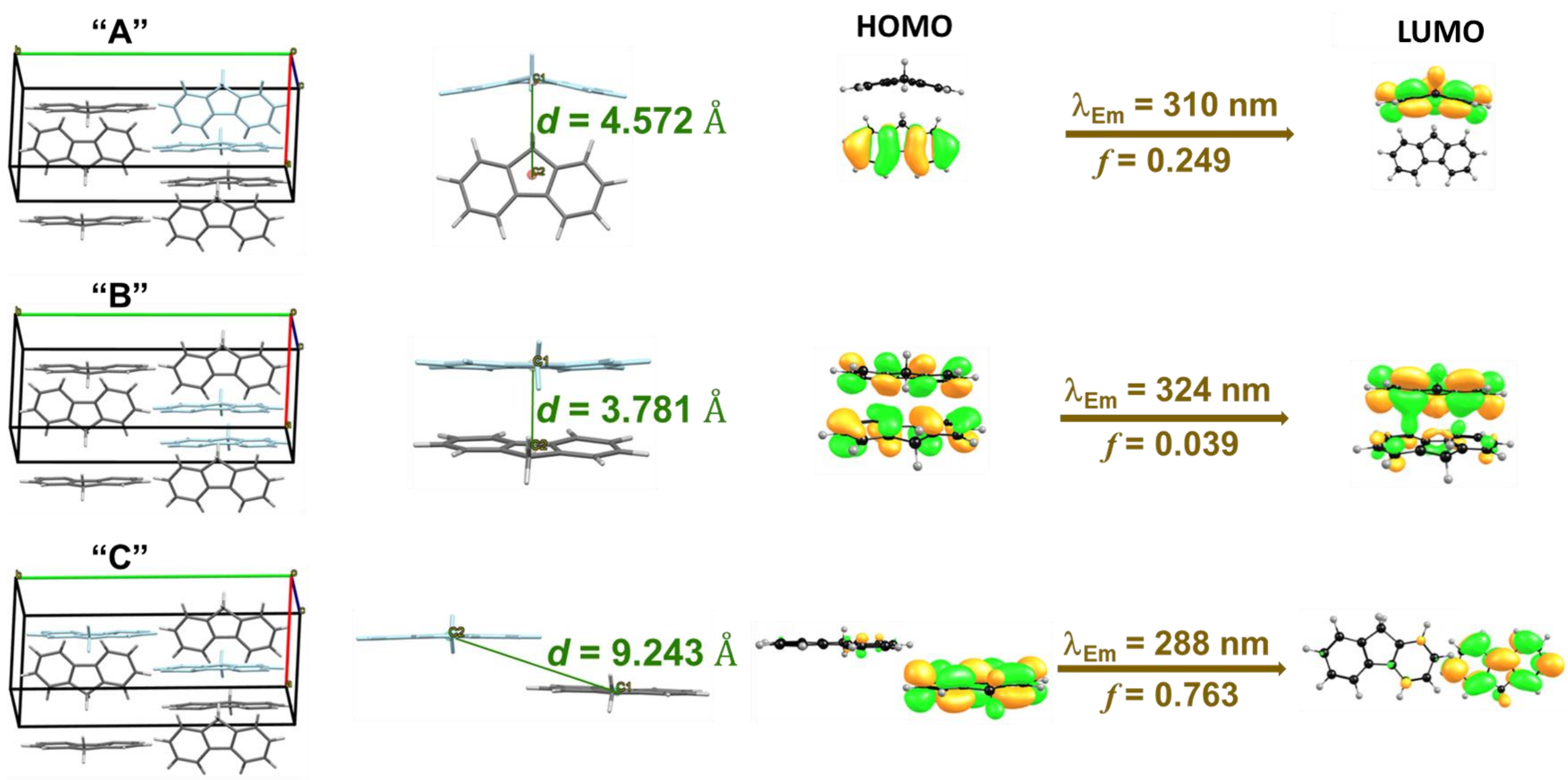

Figure S9. Natural transition orbitals (NTOs) of emission for fluorene. The dimer geometry used for the absorption NTOs was taken directly from the crystal structure, whereas the dimer geometry used for the emission NTOs was obtained from gas phase TDDFT calculations.

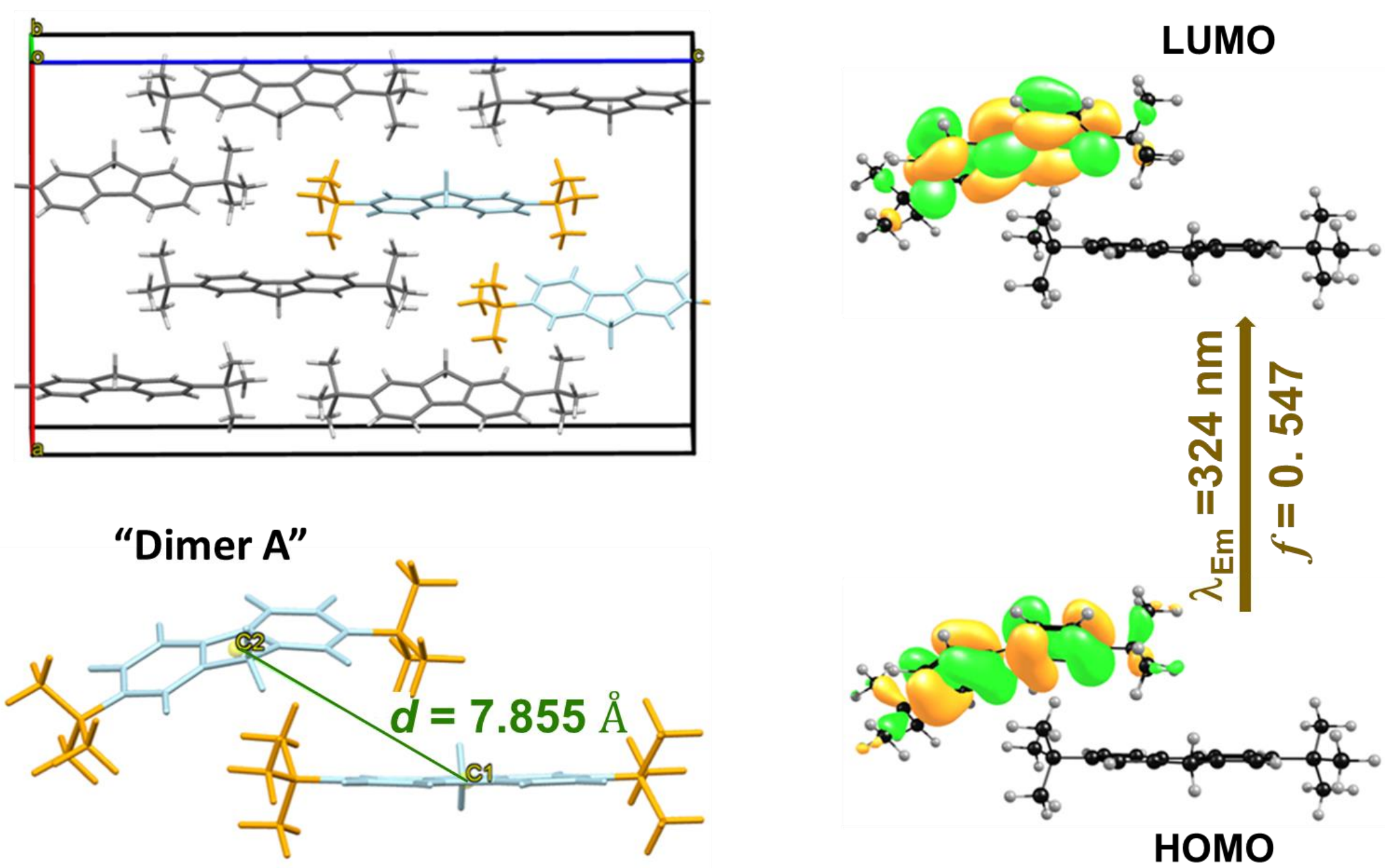

Figure S10. Natural transition orbitals (NTOs) of absorption and emission for 2,7-*dtb*F. The dimer geometry used for the absorption NTOs was taken directly from the crystal structure, whereas the dimer geometry used for the emission NTOs was obtained from gas phase TDDFT calculations.

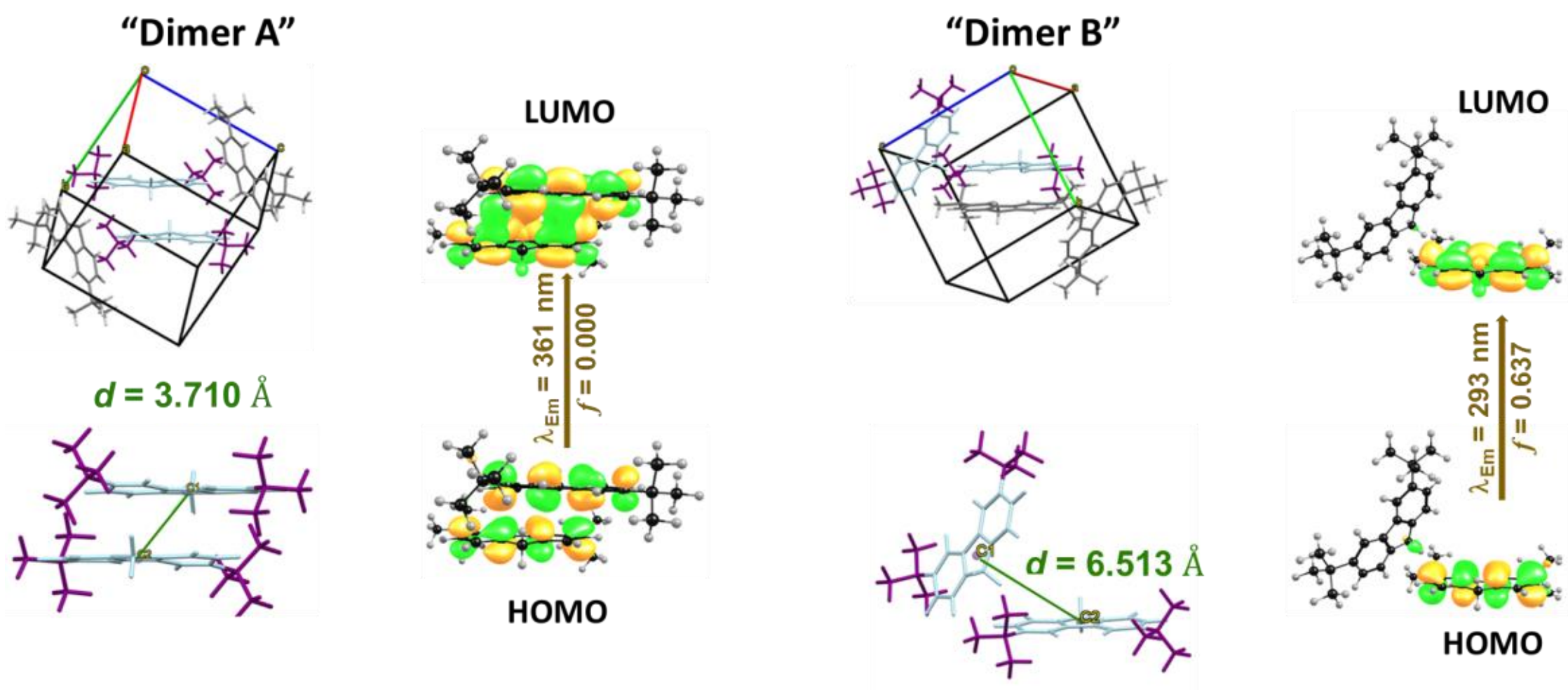

Figure S11. Natural transition orbitals (NTOs) of absorption and emission for 3,6-*dtb*F. The dimer geometry used for the absorption NTOs was taken directly from the crystal structure, whereas the dimer geometry used for the emission NTOs was obtained from gas phase TDDFT calculations.

Table S7. Theoretical emission maxima of the fluorene derivatives in the gas phase. The oscillator strength (f) is reported together with the data obtained at the DFT//LC-BPBE (ω=0.2) level of theory.

Compound	Intermolecular distance (Å)	$\lambda_{em,\ nm}$ (f)
Fluorene "A"	4.572	310 (0.249)
Fluorene "B"	3.781	324 (0.039)
Fluorene "C"	9.243	288 (0.763)
Fluorene - Antiparallel	3.152	397 (0.000)
Fluorene - Parallel	3.707	366 (0.007)
2-*tb*F - Antiparallel	3.814	344 (0.004)
2-*tb*F - Parallel	4.190	362 (0.005)
2,7-dmF - Antiparallel	3.252	387 (0.0002)
2,7-dmF - Parallel	3.164	396 (0.000)
2,7-*dtb*F - "Dimer A"	7.855	324 (0.547)
2,7-*dtb*F - Antiparallel	3.758	337 (0.000)
2,7-*dtb*F - Parallel	5.812	302 (0.567)
3,6-*dtb*F - "Dimer A"	3.710	361 (0.000)
3,6-*dtb*F - "Dimer B"	6.513	292 (0.637)
3,6-*dtb*F - Antiparallel	3.135	398 (0.000)
3,6-*dtb*F - Parallel	3.947	352 (0.005)

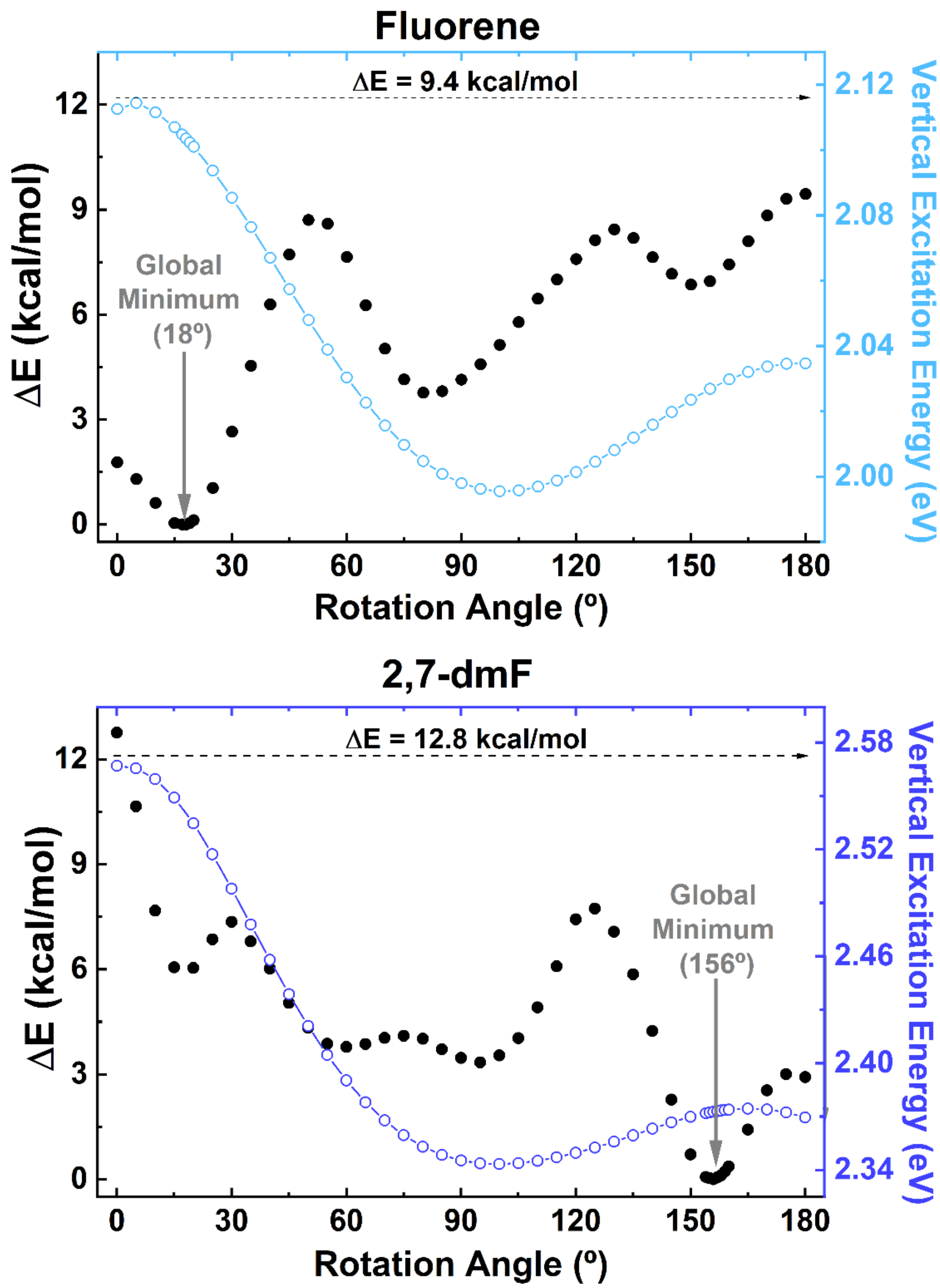

Figure S12. Constrained rotational energy scans calculated for fluorene (top) and 2,7-dmF (bottom) dimers by rotating one fluorene unit while maintaining a fixed intermolecular separation of 3.2 Å. The relative ground state energy (black symbols, left axis) is referenced to the corresponding global minimum, while the calculated vertical excitation energy (blue symbols, right axis) is plotted as a function of the rotation angle (º). The calculations show that 2,7-dimethyl substitution significantly modifies the rotational potential energy surface, leading to different preferred mutual orientations and rotational barriers, whereas the vertical excitation energy varies only slightly along the rotational coordinate.

Time-resolved fluorescence data for fluorene: diamantane mixtures

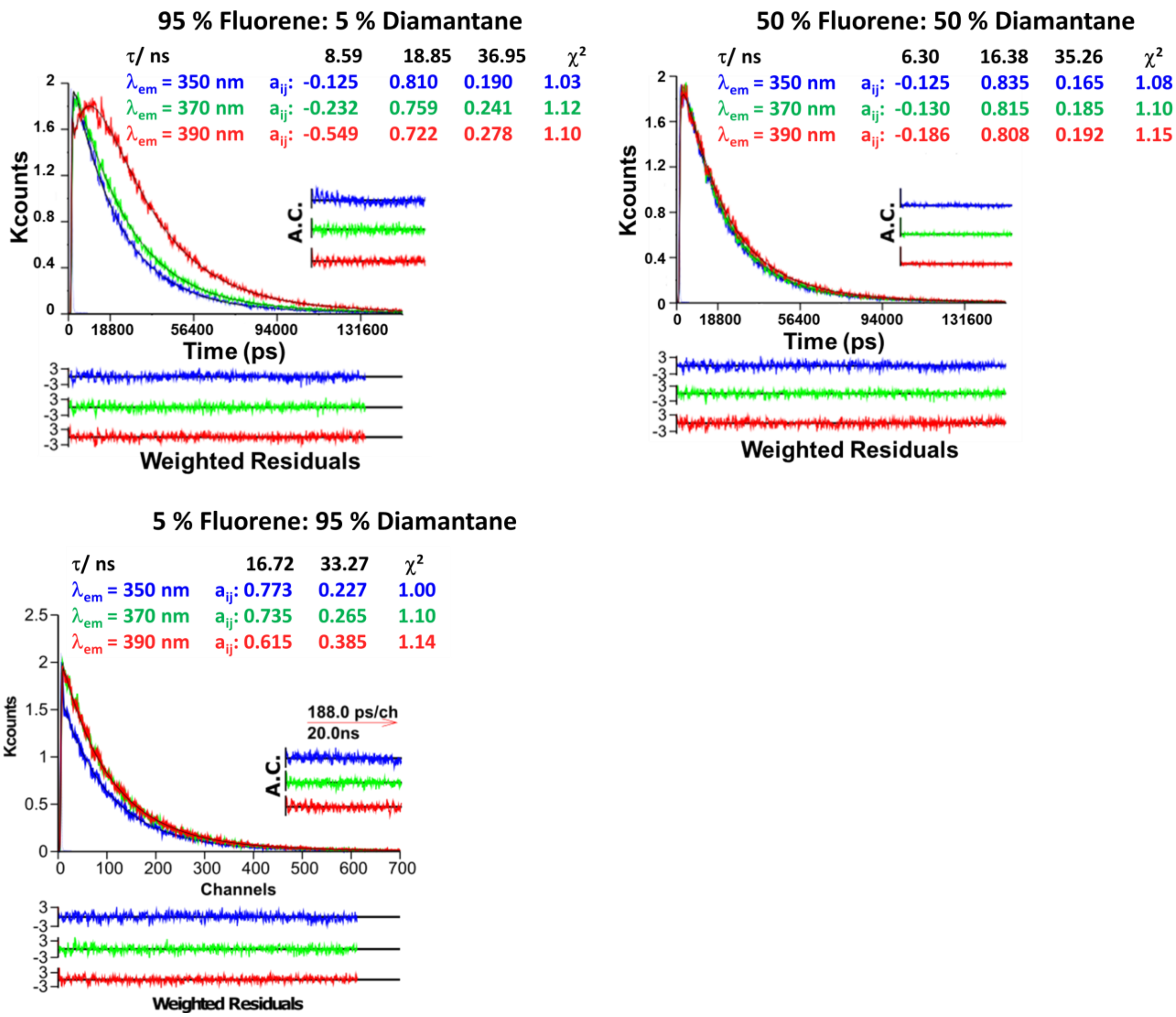

Figure S13. Room temperature time-resolved fluorescence data (lifetimes, τ_{ij}, and pre-exponential factors, a_{ij}) obtained with ns-TCSPC technique for fluorene: diamantane mixtures, collected with excitation wavelength λ_{exc} = 282 nm at different emission wavelengths.

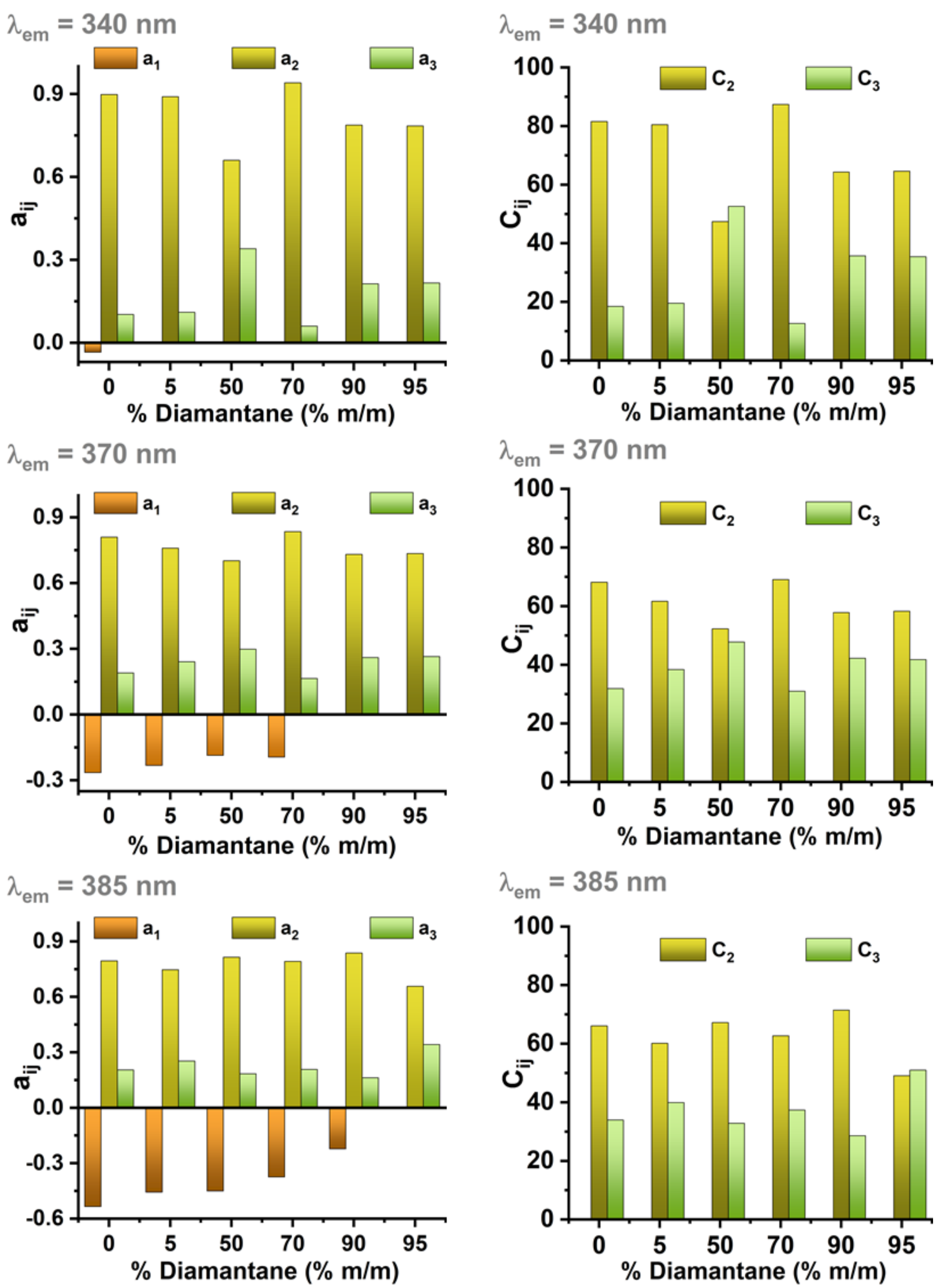

Figure S14. Room temperature time-resolved fluorescence data (lifetimes, τ_{ij}, and pre-exponential factors, a_{ij}) obtained with ns-TCSPC technique for fluorene: diamantane mixtures, collected with excitation wavelength λ_{exc} = 282 nm at different emission wavelengths.

Time-resolved fluorescence data for 2,7-dmF: diamantane mixtures

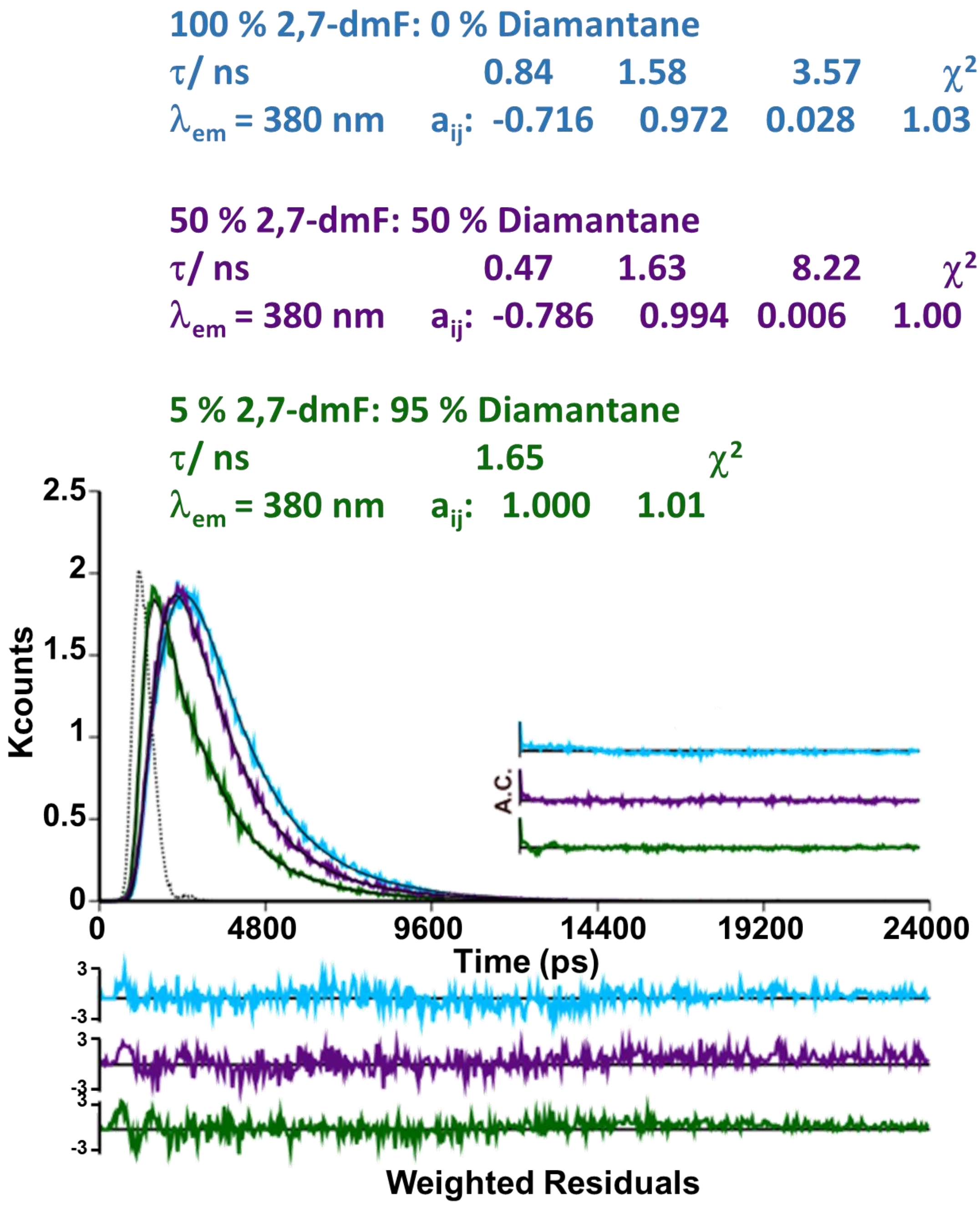

Figure S15. Room temperature time-resolved fluorescence data (lifetimes, τ_{ij}, and pre-exponential factors, a_{ij}) for 2,7-dmF: diamantane mixtures, obtained by ns-TCSPC with an excitation wavelength of λ_{exc} = 282 nm and an emission wavelength of λ_{em} = 380 nm.

Table S8. Room temperature time-resolved fluorescence data (lifetimes, τ_{ij}, and pre-exponential factors, a_{ij}) obtained with ns-TCSPC technique for 2,7-dmF, collected with excitation wavelength λ_{exc} = 282 nm at different emission wavelengths.

% Diamantane	λ_{em} (nm)	τ_1 (ns)	τ_2 (ns)	τ_3 (ns)	a_1 (C_1 %)[b]	a_2 (C_2 %)[b]	a_2 (C_3 %)[b]
**0**	350	0.84	1.58	3.57	-0.585[a]	0.983 (96)	0.017 (4)
	380				-0.716[a]	0.972 (95)	0.028 (5)
	395				-0.926[a]	0.911 (82)	0.089 (18)
**50**	350	0.47	1.63	8.22	-0.611[a]	0.999 (99)	0.001 (1)
	380				-0.786[a]	0.994 (97)	0.006 (3)
	395				-0.863[a]	0.992 (97)	0.008 (3)
**95**	350	-	1.65	-	-	1.000 (100)	-
	380						
	395						

[a]A negative amplitude corresponds to the rise-time component. [b]Data in brackets is for the fractional emissive contribution (% C).
